# Beyond monolayers: a comparative analysis of 2D cell cultures and 3D *in vitro* models as new approach methodologies

**DOI:** 10.3389/fbioe.2026.1862040

**Published:** 2026-08-24

**Authors:** Italo Rodrigo Calori, Ana Paula Pereira Guimaraes, Ananth Kumar Kammala, Lauren S. Richardson, Ramkumar Menon

**Affiliations:** Department of Obstetrics and Gynecology, Division of Basic Science and Translational Research, The University of Texas Medical Branch at Galveston, Galveston, TX, United States

**Keywords:** 3D model, bioprinting, new approach methodologies, organoid, spheroid

## Abstract

Two–dimensional (2D) cell cultures have been used as *in vitro* models in biomedical research as fundamental tools for investigating biological processes. However, their limited capacity to recapitulate the complexity of human tissues has driven the search for more physiologically relevant *in vitro* systems. In parallel, limitations in the predictive capacity, costs, and ethical concerns associated with animal models have reinforced the importance of the 3Rs principles (Replacement, Reduction, and Refinement), introduced in 1959, and more recently contributed to the emergence of New Approach Methodologies (NAMs). In this context, three–dimensional (3D) cell culture models, such as spheroids, organoids, and bioprinted models have been developed. This literature review focuses on studies that directly compare spheroids, organoids, and 3D–printed models with their 2D culture counterparts, highlighting their potential and limitations as human–relevant NAMs.

## 1 Introduction

Two–dimensional (2D) cell culture models have been widely used for over a century and remain fundamental tools in biomedical research, primarily because of their simplicity, reproducibility, and accessibility. The convenience of maintaining cells on a flat, rigid surface immersed in a culture medium has facilitated studies on cellular mechanisms (Borchardt, 1995). However, in the current stage of biomedical research, their structural and biological simplicity is increasingly recognized as a limitation. While they remain key models for studying fundamental processes, this oversimplification limits their ability to recapitulate the structural complexity, dynamic behavior, and physiological functions of native tissues (Bertsch et al., 2023). These limitations, together with the challenges associated with animal models, including their limited ability to predict human responses, ethical considerations, and economic costs, have contributed to the growing interest in the development and adoption of New Approach Methodologies (NAMs) (Zhang and Radisic, 2017; Russell and Burch, 1959). The increasing demand for NAMs has further accelerated the development of three–dimensional (3D) cell culture models in recent years (Zhang et al., 2023). This literature review summarizes and discusses comparative studies evaluating spheroids, organoids, and bioprinted systems against traditional 2D cell culture approaches as NAMs.

## 2 3D models as NAMs

NAMs encompass a diverse set of innovative technologies designed to improve the relevance of biomedical research by better representing the relevant aspects of human biology in experimental models. The concept of NAMs aligns with the principles of Replacement, Reduction, and Refinement (3Rs), introduced in 1959, by promoting the development of alternative approaches that can complement or reduce reliance on animal models. These methods include advanced 3D *in vitro* models, such as organoids, spheroids, and bioprinted systems, artificial intelligence and machine learning (AI/ML)–driven predictive tools, high–throughput screening platforms, and the integration of large–scale multi–omics datasets. These approaches expand the potential of NAMs to address complex biological issues.

In this context, organoids, spheroids, and bioprinted models have shown significant promise for the *in vitro* modeling of human tissues. Their ability to model patient–specific biology, reflect tissue heterogeneity, and simulate developmental and disease processes positions these platforms as valuable tools for bridging the gap between conventional *in vitro* models and *in vivo* studies. Consequently, these systems have gained attention as *in vitro* models for improving the translational relevance of preclinical research, enabling more human–relevant toxicity testing (i.e., developmental and reproductive toxicology [DART]), and advancing approaches for modeling physiological and pathological conditions.

## 3 Spheroids

**FIGURE 1:**
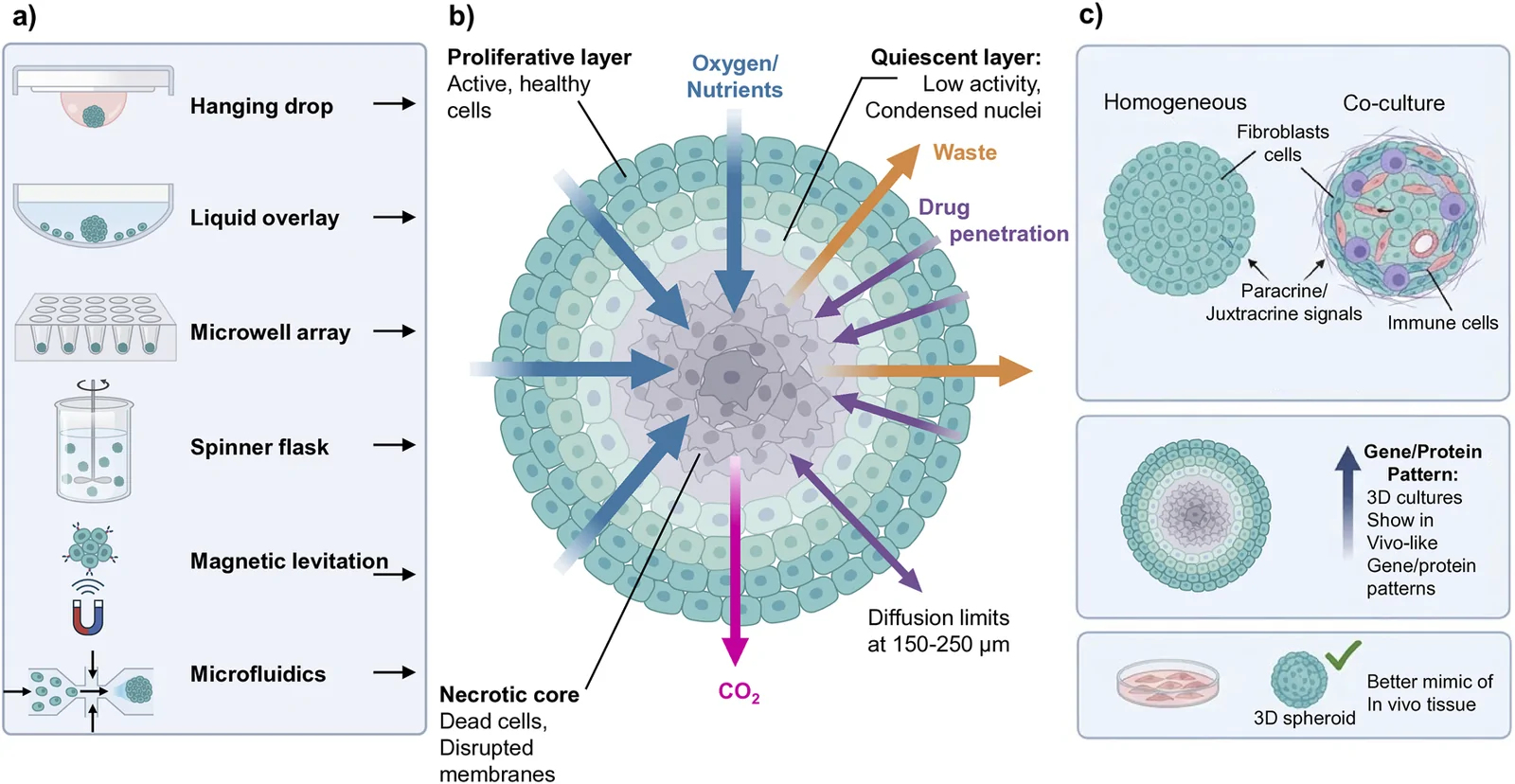
Overview of spheroid generation methods and key structural and biological features. **(a)** Common methods for spheroid generation. **(b)** Representative spheroid architecture showing proliferative, quiescent, and necrotic regions associated with gradients in oxygen, nutrients, metabolites, and drug penetration. **(c)** Biological features of spheroid models.

Spheroids are tightly compacted spherical multicellular aggregates. They are formed by the self–assembly of cells under non–attachment surface conditions (i.e., liquid/semisolid environment or solid surfaces) (Figure 1). (Chatz and inikolaidou, 2016) Owing to their 3D architecture, spheroids can replicate cell–cell and cell–matrix interactions in native tissues (Kim S. J. et al., 2020). Thus, spheroids can exhibit oxygen gradients, improved maturation, and long–term stability compared to 2D cultures (Boscaro et al., 2025; Langan et al., 2016). This architecture has also induced gene expression patterns that align more closely with *in vivo* samples than 2D models, including single-cell and bulk transcriptomic matches to primary tumors, hypoxia-stage concordance, and endothelial profiles (Zietarska et al., 2007; Däster et al., 2016; Rapp et al., 2024). It also enables the modeling of cell invasion (Boot et al., 2021) and allows the tuning of extracellular matrix deposition (Motta et al., 2026; Přikryl et al., 2025).

A broad range of strategies has been used to generate spheroids. Classical approaches include hanging–drop cultures, where multicellular aggregation occurs at the bottom of the drop, guided by gravity and surface tension, and liquid–overlay systems, in which non–adhesive coatings, such as agarose, polyacrylamide, or commercial ultra–low attachment (ULA) surfaces, support spontaneous 3D assembly (Rasouli et al., 2025; Costa et al., 2018). Additional low–complexity methods, including pellet culture, microwell arrays, and gel embedding approaches, enable rapid compaction or geometric confinement with varying degrees of control over the spheroid size (Zhang et al., 2018; Rahman et al., 2025; Su et al., 2021). Dynamic cultures, such as spinner flasks and rotating bioreactors, enhance mass transfer but often produce broader size distributions (Kriedemann et al., 2024; Massai et al., 2016; Phelan et al., 2019). Other advanced approaches use external physical forces, including magnetic levitation, liquid marbles, acoustic–based assembly, and dielectrophoretic techniques, to rapidly and reproducibly cluster cells (Park et al., 2023; Sarigil et al., 2026; Ahadian et al., 2014). Microfluidic devices are also being explored to achieve high precision in spheroid formation, as microscale channels and droplet–based systems can tightly control cell loading, micro–environmental conditions, and spheroid dimensions (Vadivelu et al., 2017).

Several models have been used to describe the formation of spheroids. The main ones include i) kinetic aggregation models (Enmon et al., 2002; O’Connor and Venczel, 2005; Enmon et al., 2001), which describe how cells in suspension collide and aggregate over time; ii) adhesion–phase models (Enmon et al., 2001; Lin et al., 2006), which, considering spheroid formation as a series of biological phases, describe how the initial contact/adhesion phase (cell–cell and cell–matrix) allows coalescence into spheroids; for heterogeneous cell populations, the differential adhesion hypothesis of Steinberg describes how differences in adhesive affinity can drive clustering and sorting of cells (Foty and Steinberg, 2005); and iii) the mechanical fusion and confinement model, which describes the later stages in which aggregates fuse into larger spheroids and, respectively, the effects of confinement (wells and microchannels) on morphology (Desmaison et al., 2018; Lugovoi et al., 2025; Susienka et al., 2016). Other models, such as the continuum reaction model, describe the transport of nutrients/oxygen and reactions within the formed spheroids (Mahmood et al., 2011).

The common key parameters in spheroid formation are size and compactness (Alves et al., 2023). These parameters depend on variables such as the cell type, formation method, and culture duration (Calori et al., 2022; Raghavan et al., 2016). Spheroids usually grow rapidly at early time points. They can be followed by continuous growth, a stabilization phase, or even a reduction in volume, which is commonly observed as a function of cell death (Calori et al., 2022). This maturation period is critical for establishing diverse characteristics, including gene expression and structural organization, resulting in tightly compacted aggregates that are mechanically stable during handling (Sargenti et al., 2023). Several spheroids demonstrated a dense inner structure (Brochado et al., 2023; Sun et al., 2022). It has also been shown that nutrient gradients are formed within spheroids (Ascione et al., 2024). Together, these morphological differences can create a more physiologically relevant environment than 2D cell cultures in several models.

Gradients of oxygen, nutrients, and drugs are established within spheroids as a function of their size. Spheroid size directly affects the molecular gradients within their structures. In tight–compact spheroids, 150–200 µm is generally accepted as the threshold for the diffusion of oxygen and other small molecules (Cui et al., 2017). Driven by a similar steric mechanism, spheroids tend to retain CO_2_ and metabolic waste in their cores (Decarli et al., 2021). As spheroids grow, their proliferative activity decreases in the core. All these parameters lead to the development of a three–layer region comprising, respectively, proliferative, quiescent, and necrotic zones in spheroids of approximately 500 µm or larger, as shown in Figure 1 (Cui et al., 2017; Decarli et al., 2021).

Spheroid characteristics can be modulated using various strategies. In terms of composition, spheroids can comprise a single cell type (using either primary or immortalized cells, or be derived from tissue crypts obtained via biopsy), resulting in a more homogeneous phase, or can be established via co–culture to generate tissue heterogeneity closer to the native tissue of interest. When different cell types are combined, they can activate the paracrine signaling, which is generally more difficult to obtain in 2D cultures (Rederer et al., 2023; Osaki et al., 2018). Stromal and endothelial cells further contribute to the deposition of extracellular matrix and to alterations in its stiffness and organization. In addition, co–culture spheroids often exhibit several advantages, such as improved proliferation and a more representative native microenvironment, and can show drug responses that more closely resemble *in vivo* patterns, including treatment resistance patterns (Guimaraes et al., 2025; Xu et al., 2013; Lazzari et al., 2018). As a result, changes in drug penetration, sensitivity, and resistance occur when fibroblasts, endothelial cells, or immune cells are present (Zietarska et al., 2007; Däster et al., 2016; Rapp et al., 2024; Boot et al., 2021). This is consistent with several transcriptomic and proteomic studies showing that 3D cultures display gene–expression patterns that align more closely with *in vivo* samples than those observed in conventional 2D models. As a result, co–culture spheroids have been shown to be more representative of the targeted native tissues than conventional 2D cultures (Motta et al., 2026; Přikryl et al., 2025; Rasouli et al., 2025). In parallel, several co–culture spheroids upregulate extracellular matrix proteins compared to 2D cultures (Selden et al., 2000; Bai et al., 2015; Qi et al., 2022; Longati et al., 2013; Härmä et al., 2010).

Its characteristics can also be modulated by strategies that permit nutrient perfusion, such as vascularization and microfluidic perfusion systems. Vascularization is frequently attempted by incorporating endothelial cells into co–culture spheroid systems. Spheroids are advantageous because they allow endogenous vascular sprouts to produce a vascular network, which is absent in 2D cultures (Laschke and Menger, 2017). Studies have shown the activation of pro–angiogenic transcriptional programs and the secretion of angiogenic factors in spheroids, highlighting the role of the 3D environment in promoting blood vessel formation (Vasilets et al., 2022).

## 4 Organoids

Organoids are self–organized, multicellular 3D culture models derived from embryonic, adult, or induced pluripotent stem cells, formed by differentiation and self–organization. Contrary to the focus on the morphology, compaction, and sphericity of spheroids, organoids aim to recreate specific organ functions, recapitulating organ microanatomy and forming microstructures analogous to tissues, with most cases exhibiting high cellular heterogeneity. Thus, organoids are currently one of the most advanced *in vitro* models for organ development and disease (Birtele et al., 2025). They have been used in a wide range of applications, including genetic diseases (Huang W. et al., 2025), immunological research (Bar-Ephraim et al., 2020), drug screening (Shin H. et al., 2025; Xie et al., 2025; Ren et al., 2023), personalized medicine (Gout et al., 2025), the reproductive system (Turco et al., 2018; Haider et al., 2018), and bacterial and viral infections (Li et al., 2023; Yu et al., 2024).

Organoid development requires longer and more complex protocols (from weeks to months) than spheroid culture, including the optimization of differentiation signals. Initially, the cells form aggregates (similar to spheroid formation) facilitated by culture conditions that promote cell–cell interactions. Subsequently, growth factors (proteins and small–molecule drugs that activate or inhibit signaling pathways) and extracellular matrix (ECM) direct lineage specification and early tissue patterning. As development progresses, morphogenetic events give rise to increasingly organized three–dimensional architectures. Ultimately, continued cellular remodeling supports tissue maturation, characterized by the emergence of specialized cell populations and tissue–specific features that mimic native tissues, as assessed by cellular composition, gene expression, and biological characteristics that display the key functional, structural, and biological complexity of the specific native tissue or organ of interest.

**FIGURE 2:**
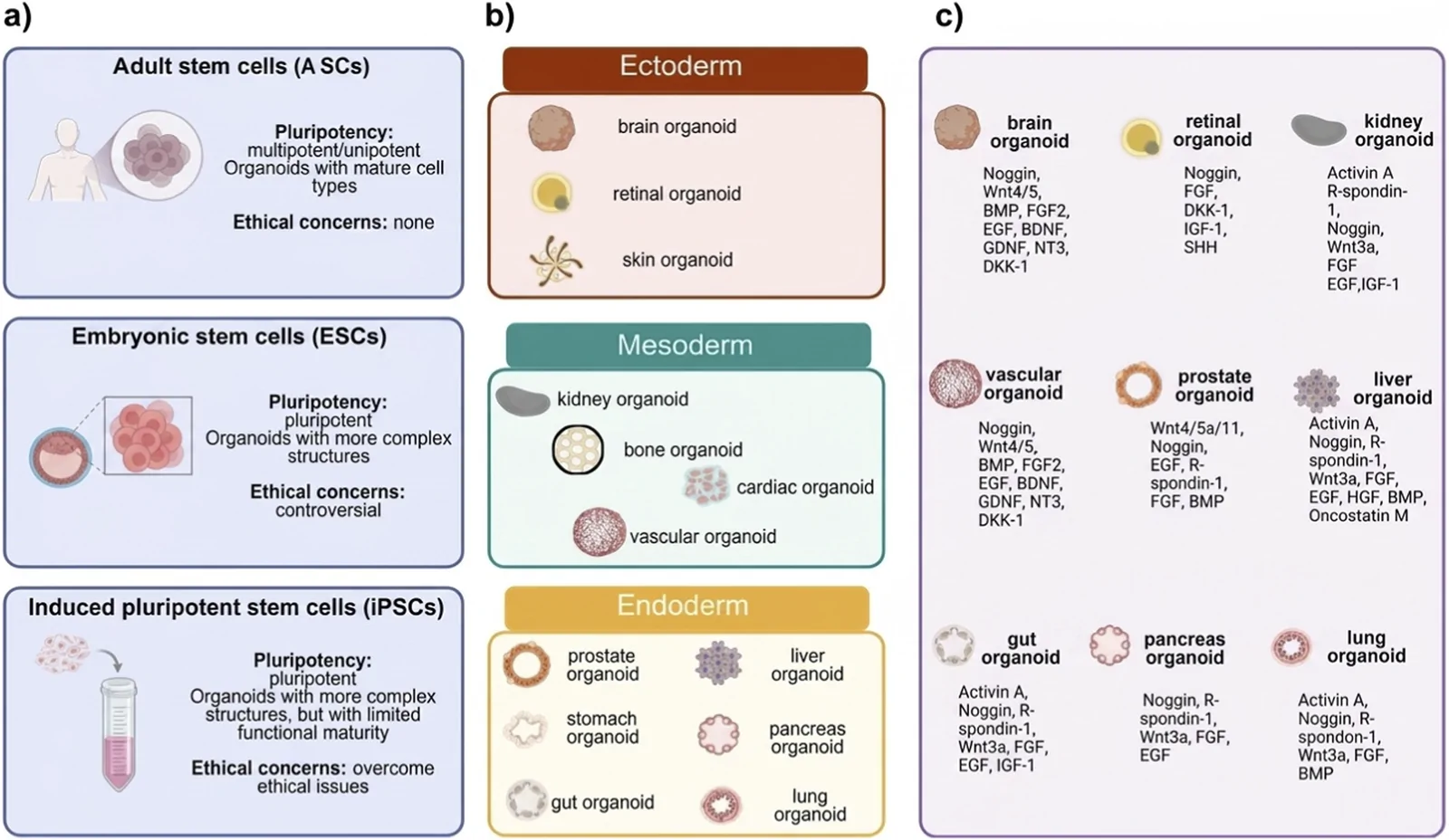
Cell sources, germ-layer classification, and key signaling cues for organoid generation. **(a)** Major cell sources used for organoid development, including adult stem cells (ASCs), embryonic stem cells (ESCs), and induced pluripotent stem cells (iPSCs). **(b)** Representative organoid types derived from the ectoderm, mesoderm, and endoderm. **(c)** Examples of key growth factors and signaling molecules used to promote organoid differentiation, expansion, and tissue-specific patterning.

The source of cells for organoid fabrication generally includes pluripotent stem cells (PSCs) and adult stem cells (ASCs) (Figure 2). PSCs can be human embryonic stem cells (hESCs) or induced pluripotent stem cells (iPSCs). hESCs are derived from blastocysts, and iPSCs are produced by reprogramming somatic cells, with iPSCs being able to differentiate into any cell type from the three germ layers (ectoderm, mesoderm, and endoderm). ASCs retain the ability to differentiate and can be used to generate tissue–specific organoids derived from their tissue of origin. ASCs are obtained from biopsy samples or surgical resections of human adult tissues and have either multipotent or unipotent differentiation potential, allowing them to generate a limited range of cell types within their tissue of origin. They can be expanded *in vitro* under defined culture conditions while maintaining controlled self–renewal and differentiation (Boscaro et al., 2025). As ASCs are derived from patients, they are crucial for personalized medicine. It is worth noting that the use of human ASCs does not raise ethical concerns related to embryo destruction. Instead, the main ethical considerations are informed patient consent for tissue donation and data privacy.

## 5 Bioprinting

### 5.1 Principles

Bioprinting is an additive manufacturing–based technology that enables the automated fabrication of biological constructs through the digitally controlled deposition of materials containing living cells in 3D architectures. Compared with 2D cell cultures, bioprinting is technically more advanced in terms of constructing specific 3D structures that better replicate organ architecture, tissue hierarchy, and heterogeneity. Its technical advantages are partly due to the use of computer–aided design (CAD) and clinical imaging (e.g., computed tomography) data to recreate, modify, and optimize the desired 3D structure. This enables researchers to mimic the hierarchical structure and improve architectural accuracy in terms of geometry and cellular organization (Shahabipour et al., 2022). Thus, bioprinting has further expanded the ability of 3D *in vitro* models to mimic the tissue environment, extracellular matrix, and barriers such as the blood–brain barrier, while allowing precise control of experimental conditions. In addition, by controlling parameters such as hydrogel viscoelasticity and bioink composition, time–dependent shape transformations of engineered tissues can be programmed, a concept known as next–generation 4D bioprinting (Pramanick et al., 2025).

One of the most critical steps for the success of printing is the development and optimization of bioinks with biocompatible mechanical properties that favor long–term cell functionality (Qian et al., 2019; Akter et al., 2025). In early bioprinting, an important challenge is ensuring permeation and waste product removal in thick tissues, which can result in extensive cell death. Currently, perfusable bioprinted models have been fabricated using a range of techniques, including the use of sacrificial inks and volumetric bioprinting techniques. In addition, novel approaches have been developed to allow the fabrication of perfusable vascularized constructs using sacrificial–free direct ink writing (Wu et al., 2024a). Another challenge is cell homogenization, particularly for bioinks with high viscosity.

As bioprinting is a relatively recent technology, it is constantly being improved and optimized. The lack of standardization of the homogenization process in the literature, along with the increase in the number of research groups migrating to bioprinting, has resulted in low reproducibility and led to either heterogeneity or excessive cell death. Automated processes have been proposed to improve some stages of bioink preparation, enhancing the reproducibility and reliability of the bioprinting process (Wu et al., 2024b). Supplementary Table S3 summarizes the main differences observed between bioprinted models and 2D cell cultures.

### 5.2 Techniques

A range of bioprinting techniques allows the precise deposition of biological materials into 3D structures, differing in the type of biomaterials used, precision, time, and cost, among others. The most common is direct ink writing (DIW), an extrusion–based bioprinting technique. DIW, particularly continuous ink extrusion, has been extensively used for bioprinting, typically because of its affordability, ease of use, potential for scaling up, and speed (Saadi et al., 2022). It can print complex biological structures at the meso–and microscale using a wide range of biomaterials as they exhibit suitable rheological behavior (Saadi et al., 2022).

Other common techniques include stereolithography (SLA), and digital light processing (DLP), both of which are laser–assisted printing techniques. In parallel, volumetric bioprinting technologies have gained increasing attention for the fabrication of intricate and perfusable biostructures. These technologies have the advantage of not using a layer–by–layer approach but instead crosslinking the entire construct simultaneously, significantly reducing the printing time. They also allow the production of viscosity gradients through printing, which is useful for producing interfaces and other complex native tissue equivalents. A detailed description of each technique is beyond the scope of this review, and several excellent papers have already provided comprehensive overviews of these methods and can be consulted for further information (Starly and Shirwaiker, 2015; Wu et al., 2023; Picad et al., 2025; Xu et al., 2025).

## 6 Comparative analysis between 3D models and 2D cultures

This section provides a comparative analysis of 3D *in vitro* models and conventional 2D cell cultures. It synthesizes the most recent findings on spheroids, organoids, and bioprinting compared with 2D cultures (or similar models) to better elucidate their distinct advantages and limitations.

### 6.1 Spheroids versus 2D culture

**FIGURE 3:**
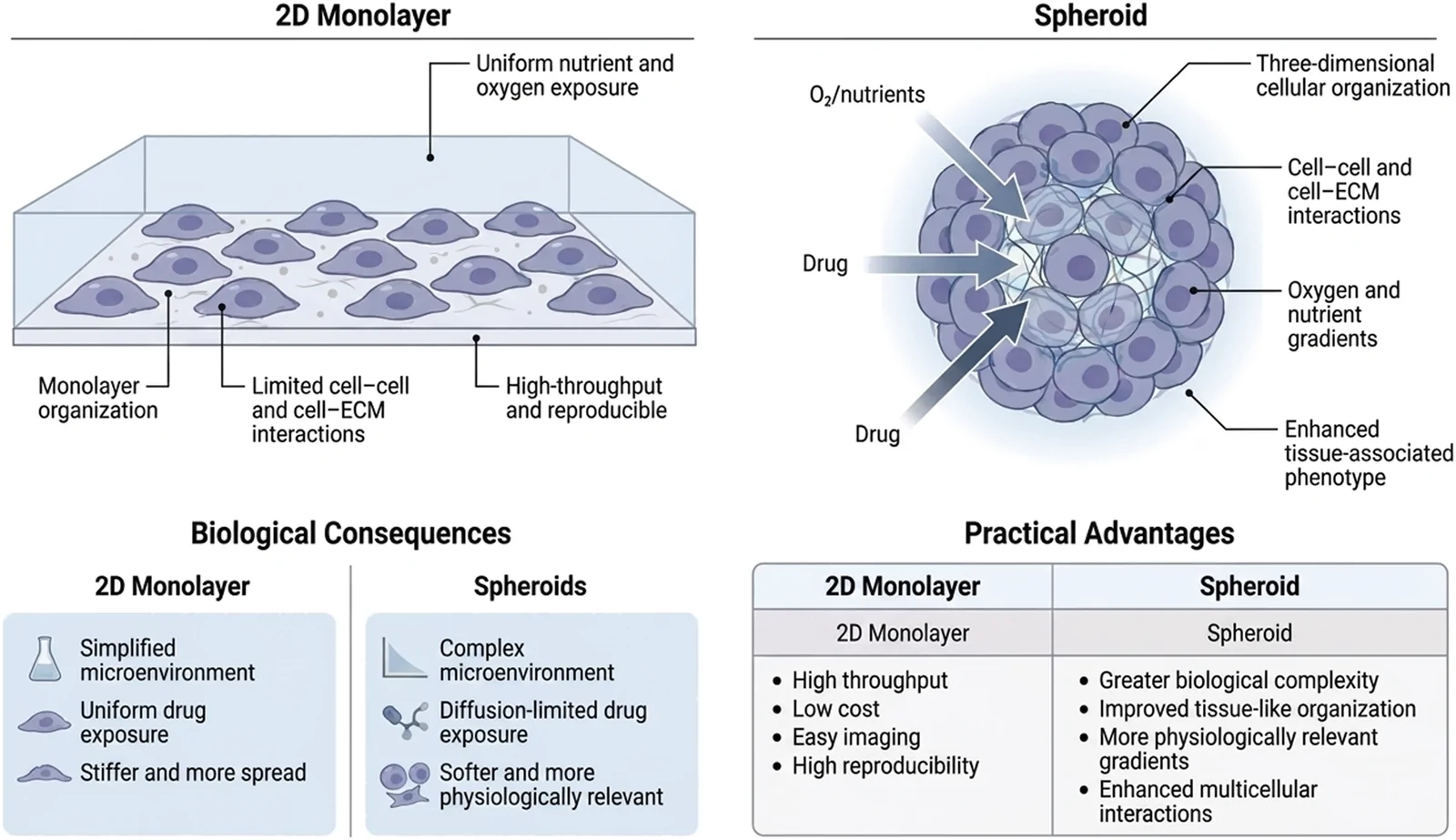
Comparison of 2D monolayer and spheroid culture models. Schematic overview of the principal biological and experimental differences between 2D and 3D culture systems, emphasizing microenvironmental complexity, transport limitations and physiological relevance.

The literature comparing spheroid models and 2D cell cultures has shown differences in morphology, physiological gradients, metabolism, transporter function, gene expression, secretory profiles, toxicological response, and long–term stability. Supplementary Table S1 summarizes the differences between spheroid models and 2D cell cultures. Direct comparisons across multiple tissue types have shown that spheroids can better reproduce tissue–specific functions, including healthier adipokine profiles, hepatic albumin/urea secretion and CYP activity, renal transporter function, cardiac tissue–like expression, and trophoblast invasion more closely resembling placental tissue (Seo et al., 2025). The key differences between spheroids and 2D models are summarized in Figure 3.

Compared with 2D cultures, spheroids demonstrate improved tissue–like organization (Liszewski et al., 2024; Beauchamp et al., 2020; Mizuguchi et al., 2021). In adipose spheroids, larger lipid droplets and a higher proportion of unilocular adipocytes are formed, in contrast to 2D cultures, in which adipocytes are more multilocular (Liszewski et al., 2024). In renal spheroids, adult renal progenitor cells self–organized into long tubule–like structures that are absent in 2D cultures (Giannuzzi et al., 2025).

Placental extravillous trophoblast spheroids exhibit upregulation of genes and processes critical for placental invasion, including EMT, angiogenesis, and general invasion markers (Wong et al., 2019). In adipose models, spheroids show increased expression of LIPE, FABP4, and ADIPOQ expression. Hepatic and cardiac organoids have shown global transcriptional reprogramming (Sun et al., 2024).

Spheroids also demonstrate enhanced secretory profiles and reduced pro–inflammatory cytokine production compared with 2D cultures. For example, Liszewski et al. (2024) showed that adipose spheroids secreted more adiponectin and leptin while reducing the levels of IL–6, IL–8, and MCP–1 (Liszewski et al., 2024). Hepatic spheroids display increased albumin and urea secretion compared with 2D cell culture (Seo et al., 2025). They also exhibited higher expression and activity of cytochromes P450, improved drug–metabolizing function, increased triglyceride accumulation, and enhanced metabolism of exogenous free fatty acids compared with 2D cultures (Leite et al., 2019; Saleh et al., 2025).

**FIGURE 4:**
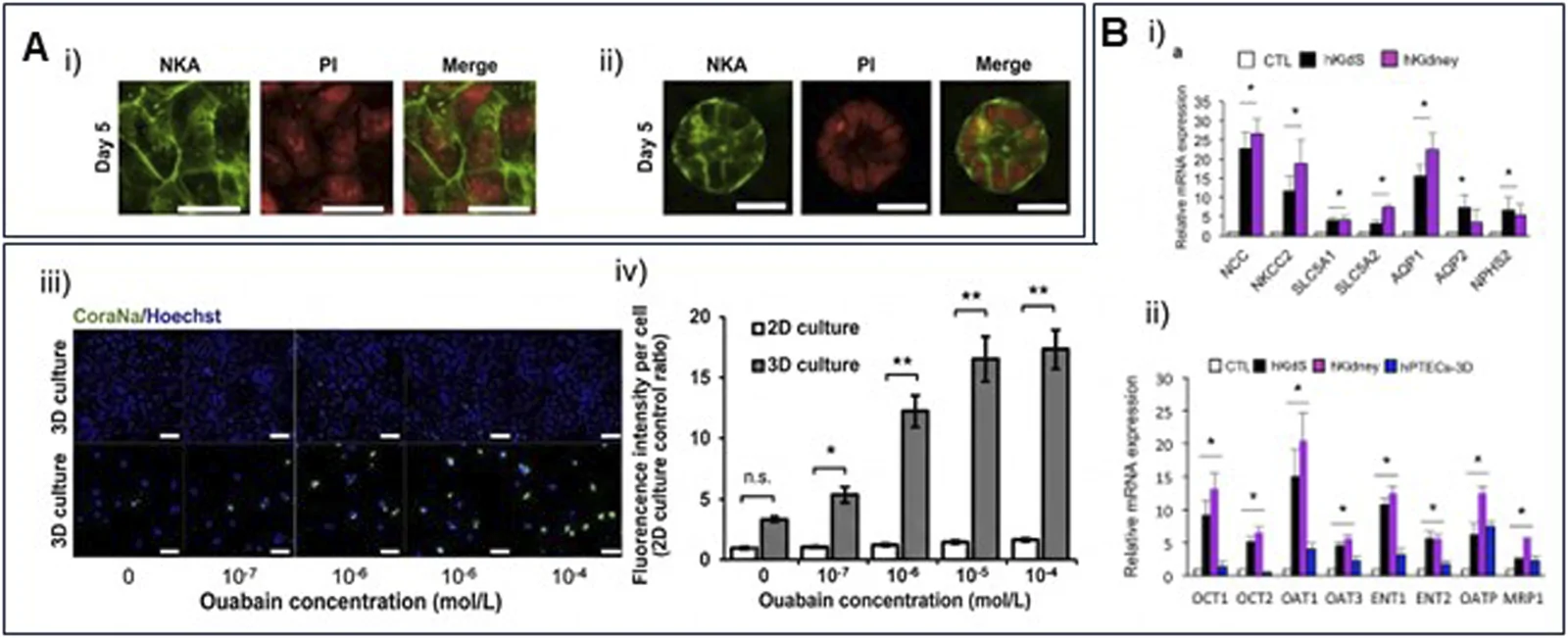
Structural, molecular and functional advantages of renal spheroid cultures over 2D cultures. **(A)** Three-dimensional culture of human renal proximal tubule epithelial (HK-2) cells promotes apical–basal polarity, illustrated by Na^+^/K^+^-ATPase (NKA) localization (i,ii), and restores functional Na^+^ transport, as assessed by CoroNa Green fluorescence following ouabain treatment (iii,iv). **(B)** Human kidney spheroids (hKidS) and hPTECs-3D spheroids show increased expression of renal lineage markers (i) and uptake/efflux transporters (ii) compared with 2D controls (2D CTL), with profiles approaching those of human kidney tissue (hKidney). Adapted from Mizuguchi et al. (2021) and Kang et al. (2019), with permission.

Spheroids have been extensively used in renal models (Guimaraes et al., 2025). Studies of renal proximal tubule (RPTEC) cells have shown that RPTEC spheroids maintain higher and more stable mRNA levels of key drug transporters, including OAT1, OCT2, MATEs, and P–gp, than 2D RPTEC cultures (Arakawa et al., 2024). Transporter expression also approached the levels observed in mouse kidney lysates (Figures 4Bi, ii). (Kang et al., 2019) Regarding the predictive ability of 3D and 2D models for drug–induced kidney injury, a recent study showed that 3D–RPTECs exhibit higher predictive performance than 2D–RPTECs and HK–2 cells. The ROC–AUC values for 3D–RPTECs were 0.941 (day 7 culture) and 0.933 (day 28 culture) compared with 0.861 for 2D–RPTECs and 0.584 (for day 1)/0.776 (for day 3) and HK–2 cells (Arakawa et al., 2024). Mizuguchi et al. (2021) demonstrated functional Na^+^ transport in 3D HK–2 spheroids (Figure 4A). Although the expression of Na^+^/K^+^–ATPase α1 was detected in both 2D and 3D HK–2 cells (Figures 4Ai,ii), the 3D–cultured cells exhibited significantly greater Na^+^ retention than the 2D–cultured cells, particularly with increasing ouabain concentrations (Figures 4Aiii,iv).

Direct comparisons of spheroids and 2D cultures have demonstrated context–dependent responses in terms of drug sensitivity. Although spheroid architecture may reduce drug penetration and cytotoxicity, enhanced sensitivity has also been reported, depending on the drug mechanism and spheroid characteristics. For example, HepaRG spheroids have been shown to exhibit genotoxicity at lower concentrations of nitrosamine than 2D cultures, indicating that these spheroids display greater sensitivity to such drugs (Seo et al., 2025).

Although technically more demanding, spheroids are often longer–term, more mature, and more stable than 2D cultures (Zh et al., 2021). They generally maintain secretory function, transporter expression, and enzyme activity over a more extended culture period (weeks) than 2D cultures. For example, it has been demonstrated that 3D hepatocyte spheroids retain CYP activity for longer durations than 2D hepatocyte cultures (Jamshed et al., 2024). A comparative analysis of 3D culture methods for human HepG2 cells using different scaffold materials found that the increased metabolic capacity observed in 3D cultures is primarily due to prolonged cultivation time rather than the 3D environment (Figure 4A). (Luckert et al., 2017) In parallel, introducing a 3D phase into an alternating 2D/3D strategy reduced senescence while preserving the anti–inflammatory activity of placenta–derived MSCs compared with 2D expansion only (Pan et al., 2025).

The differences between spheroid and 2D models are influenced by factors affecting their performance, reproducibility, and translational relevance. Unlike 2D cultures, spheroids require more time to establish a stable three–dimensional architecture, leading to differences in cellular phenotypes prior to treatment (Hirschhaeuser et al., 2010; Kunz-Schughart et al., 2004). In addition, factors such as the cell source, spheroid size, and matrix composition contribute to variability in drug responses and reproducibility (Vinci et al., 2012; Breslin and O'Driscoll, 2016; Friedrich et al., 2009).

Collectively, these studies demonstrate that spheroids can improve tissue–specific architecture and function over conventional 2D cultures, although their performance remains dependent on variables, such as cell type, maturation state, and experimental design.

### 6.2 Organoids versus 2D culture

Several studies have demonstrated the advantages of organoids over 2D cultures in a range of areas, including tissue–like architecture and function, improved drug–response prediction, enhanced health and disease modeling, and transcriptomes closer to those of native tissues. Supplementary Table S2 summarizes the main differences between organoid models and 2D cell cultures. Organoids produce layered structures that resemble *in vivo* microanatomy, whereas differentiated 2D cultures generally yield simpler sheet architectures (Nickerson et al., 2021). For example, while most 2D neural cultures need neuroexcitatory neurotransmitters such as glutamate to achieve coordinated activity, human iPSC–derived cortical organoids showed spontaneous synchronized neural activity without the presence of synchronizing stimuli (Trujillo et al., 2019). In reproductive biology, organoids have produced syncytiotrophoblast phenotypes in the absence of exogenous induction compounds, indicating spontaneous syncytia formation (Hawkins et al., 2025). These self–organize into multinucleated syncytiotrophoblast inner layers, and the secretion of high β–hCG levels mimics important trophoblast functions such as extravillous trophoblast invasion (Hawkins et al., 2025). Another study showed that term organoids with outward–facing syncytiotrophoblast layers exhibit physiological polarity, characteristic of chorionic villi *in vivo* (Yang et al., 2024). The electrophysiological activity of organoids demonstrated well–defined and sustained action potential waves that have the potential to study disorders associated with cardiac malformations, a capability crucial for reducing, refining, and ideally replacing animal use in research (Lewis-Israeli et al., 2021).

While renal 2D cultures provide a cell organization restricted to lateral cell–cell interactions, kidney organoids have been produced with complex nephron–like structures comprising approximately five nephron–like segments, including proximal tubular, distal tubular, and podocyte populations arranged in a distal–to–proximal manner. In pulmonary tissue, organoids have allowed single hAT2 cells to self–organize into alveolar–like heterogeneous 3D structures, including folded and cystic morphologies with lamellar bodies.

Some organoids have produced transcriptomic profiles closer to native tissues, including human fetal hearts, than 2D differentiated cultures (Lewis-Israeli et al., 2021). 2D cell cultures commonly lack robust endothelial/epicardial transcriptional signatures (Kerr et al., 2021). Heart organoids can present multi–cell type complexity with developmental and maturation characteristics close to embryonic fetal hearts (Lewis-Israeli et al., 2021; Kerr et al., 2021; Israeli et al., 2020). The same advantage has been observed for extracellular matrix gene expression (Israeli et al., 2020; Lewis-israeli et al., 2022). For example, hepatoblast organoids show glycogen storage, urea production, indocyanine green uptake and release, and drug–metabolism and detoxifying functions (Wu et al., 2025). Interestingly, several bioenergetic features comparable to adult liver tissue–derived hepatic organoids, such as mitochondrial oxidative phosphorylation activity and ATP production, were significantly higher in organoids than in 2D cell cultures (Mun et al., 2019).

Expandable liver organoids have addressed the limitations of the limited proliferative capacity of equivalent monolayers, demonstrating long–term expansion and self–renewal capacity in both suspension and Matrigel, and passaging over several months while maintaining normal karyotypes (Mun et al., 2019; Hendriks et al., 2021).

Pancreatic adipose tissue organoids recapitulate native tissue phenotypes and functional responses better than conventional monolayers (Lorza-Gil et al., 2025). They exhibit elevated mRNA levels of key adipogenic markers, comparable to monolayer adipocytes, and reach levels similar to those of *in situ* adipocytes (Lorza-Gil et al., 2025). In terms of long–term proliferation and viability, while monolayer cells exhibited significant proliferation rates at confluence, no Ki67^+^ cells were detected in organoids, demonstrating proliferation suppression, a characteristic of mature adipose tissue (Lorza-Gil et al., 2025). Functionally, adipose organoids displayed more robust lipolytic responses, with significantly higher expression of beta–adrenergic receptors than monolayers, and produced greater free fatty acids upon stimulation compared with monolayers (Lorza-Gil et al., 2025). They also demonstrated significant insulin–mediated suppression of basal lipolytic rates, which was not observed in the 2D cultures (Lorza-Gil et al., 2025).

The magnitude of 3D tissue compaction can influence drug penetration and efficacy; the more compact the tissue, the lower the drug penetration (Alves et al., 2023). Intriguingly, some studies have reported that 3D organoids are even more sensitive to certain drugs than monolayers, in the opposite direction of dimension/compaction (Mun et al., 2019; Xu et al., 2024). For example, the IC_50_ (half-maximal inhibitory concentration) for trovafloxacin in organoids was 2.7 µM, significantly lower than 141 µM in 2D MHs (Mun et al., 2019).

The matrix composition and density can also affect drug penetration and diffusion, leading to pharmacological responses that differ from those observed in conventional 2D systems. As a result, organoids may exhibit either increased resistance or enhanced sensitivity to therapeutic compounds compared to 2D cultures (Schutgens and Clevers, 2020; Fatehullah et al., 2016). The direction of this response depends on several factors, including organoid maturity, matrix composition, multicellular interactions, drug mechanism of action, and the experimental endpoint being evaluated (Vlachogiannis et al., 2018; Kim J. et al., 2020). Undetected in 2D cultures, decreases in spontaneous neural activity were observed in response to ivermectin, palonosetron, and pyrimethamine (Slavin et al., 2021). Conversely, mefloquine and sertraline produced the opposite effect on spontaneous neural activity (Slavin et al., 2021). A kidney organoid also demonstrated cisplatin toxicity, which could not be detected in a homogeneous 2D culture (Kang et al., 2019). For the liver, human hepatocyte–like liver organoids better predict mitochondrial toxicity at low doses of trovafloxacin than 2D models, indicating that hepatocyte–like liver organoids maintain their native drug–metabolizing activity and susceptibility to toxicity as a viable *in vitro* model for evaluating drug toxicity (Mun et al., 2019).

### 6.3 Bioprinting versus 2D culture

Bioprinting has significantly advanced the development of 3D *in vitro* models in various fields, as it allows the precise spatial deposition of biological matter to fabricate intricate *in vitro* models (Moghimi et al., 2023). Bioink composition plays a central role in determining cell viability, proliferation, phenotype, and drug sensitivity, as differences in matrix composition and mechanical properties directly influence cell behavior (Murphy and Atala, 2014). Through the controlled placement of multiple cell types and biomaterials, bioprinting can reproduce aspects of tissue organization and cellular crosstalk observed *in vivo* (Murphy and Atala, 2014; Zhang et al., 2016).

Several technical variables affect model performance and reproducibility. Printing parameters, bioink properties, construct architecture, and post–print maturation significantly influence cell distribution, viability, and functional outcomes. Consequently, the differences between bioprinted and 2D models reflect not only dimensionality but also matrix composition, tissue organization, maturation state, and manufacturing conditions (Mandrycky et al., 2016).

Bioprinted cancer models provide a complex and physiologically relevant environment for assessing cancer therapies. These models enable direct visualization and quantification of cell infiltration into solid tumor structures, which 2D models cannot achieve. Studies have shown critical differences in Chimeric Antigen Receptor T–cell (CAR–T) cell therapy evaluation compared with monolayers, in which bioprinted tumors offer a range of advantages in advanced therapy studies, including CAR–T cell therapy (Grunewald et al., 2021). For example, the lower and delayed cytotoxicity and interferon–gamma release observed in the 3D model suggest that monolayers may overestimate CAR–T cell efficacy, making 3D models a superior tool for preclinical studies (Grunewald et al., 2021). Other differences between 2D and 3D models for CAR–T cell therapy studies include higher CAR–T cell activation in the 3D model compared to that in 2D cultures (Grunewald et al., 2021).

One of the most remarkable differences is the significant reduction in the sensitivity of chemotherapeutic agents observed in 3D models. In a 3D–printed lung cancer model, the gemcitabine concentration required to induce cell death in 3D cultures was 1000–fold higher than that in monolayers (Wu et al., 2021; Mei et al., 2023). Similarly, bioprinted breast cancer models showed approximately 70–fold higher IC_50_ values than 2D cell cultures (Blanco-Fernandez et al., 2022). In another study, a neuroblastoma IMR–32 cell–based bioprinted model showed higher resistance to panobinostat than 2D cultures (Wu et al., 2021). Furthermore, primary human kidney fibroblasts were significantly more resistant to panobinostat in both 2D and 3D cultures compared with IMR–32 cells (Wu et al., 2021). Exceptions still exist. It has been shown that a bioprinted melanoma model responded similarly to 2D cultures when treated with etoposide (De Villiers and Du Plessis, 2023).

In terms of the physical steric effect, the dense network between the hydrogel and cells in 3D models, cell–ECM and cell–cell interactions (Blanco-Fernandez et al., 2022), has been described as a critical factor. This cytostatic effect is considered closer to the biological dynamics and more accurately reflects the drug resistance observed in *in vivo* tumors than in 2D cultures (due to the immediate drug–cell contact in monolayer models) (Mei et al., 2023; Blanco-Fernandez et al., 2022). Gene and protein expression related to drug resistance is another important modulator (Blanco-Fernandez et al., 2022).

Gene and protein expression is frequently modulated in 3D models compared with 2D models. Hypoxia–related genes were upregulated in endothelial cells, astrocytes, and genes associated with endothelial–mesenchymal transition in bioprinted models, indicating angiogenesis (Tung et al., 2024). The same study showed that a glioblastoma (GBM) subtype in the 3D–printed samples had the highest expression of clinically relevant genes compared with clinical GBM samples (Tung et al., 2024). According to studies on drug-resistance proteins, multidrug resistance proteins (ABCC1 and ABCG2) are more highly expressed in bioprinted hydrogels than in 2D cultures. (Kim S. J. et al., 2020), (Blanco-Fernandez et al., 2022) The upregulation of HSP90AA1 and HSP90AB1 (chaperone heat shock proteins) in 3D cultures is described as the reason for the higher survival rate in the presence of doxorubicin in 3D models. Invasion markers showed upregulation in bioprinted liver carcinoma models, in contrast to physiological–like controls, indicating that 3D models may be a more relevant platform for studying such tumors *in vitro* compared with 2D cultures (Guagliano et al., 2022).

2D and bioprinted models often demonstrate different inflammatory responses. A recent study showed that the significant increase in inflammatory markers such as IL–6, IL–8, and IL–1β in 2D culture was significantly reduced in bioprinted vascular smooth muscle cell models (Gold et al., 2021). Significantly reduced IL–6 levels were also demonstrated when stimulated with lipopolysaccharide (LPS) compared with 2D cultures. Both cases suggested less exaggerated and more physiologically relevant proinflammatory responses in the 3D environment (Sullivan et al., 2023). However, upregulation of inflammation–related genes has also been described in bioprinted models, including in a human neurovascular unit bioprinted for glioblastoma (Tung et al., 2024).

Physiological and transcriptomic differences have been observed in bioprinted models compared with 2D cultures. In a bioprinted human neurovascular unit model for glioblastoma, the predominant endothelial cell populations (EC1 and EC2) observed in 2D were absent in the 3D NVU, where an EC3 population dominated (Tung et al., 2024). This EC3 population showed high expression of genes associated with angiogenic tip cells, clinical capillaries, and blood–brain barrier–related functions, which is indicative of the greater physiological relevance of the 3D model compared with the 2D cultures. Similar to the endothelial cell population, a dominant AC4 astrocyte subcluster was detected in the bioprinting, which was absent in 2D. This population showed high expression of mature astrocyte end–foot–related genes, indicating a more physiologically relevant astrocyte type in the bioprinted model (Tung et al., 2024).

The structural and functional complexity of the central nervous system, including neural network organization, myelination, intricate neuron–glial interactions, and selective blood–brain barrier organization, is beyond the capabilities of current 2D models. In contrast, 3D models allow for the growth and extension of neural cells in a three–dimensional space (Chen et al., 2024). This enables more diverse synaptic connections, forming neural networks not only on a single plane but also radially, permitting a better simulation of the functional structure of cortical layers (Chen et al., 2024).

Neuronal differentiation was enhanced in 3D models, with a significant increase in the percentage of cells positive for the mature neuronal marker MAP2 compared to 2D cultures. Moreover, iPSC–derived neurons cultured in 3D exhibited a significant increase in the number of spontaneous calcium spikes per neuron compared to those in 2D cultures, suggesting more complex and physiologically relevant neuronal function, consistent with the improved structural and network complexity afforded by 3D models. In bioprinted models of Alzheimer’s disease, significant morphological differences, including a more structured cytoskeletal organization and extended 3D neurite networks, have been described (Chen et al., 2024). In another case, a bioprinted epilepsy model showed electrophysiological signals that more closely resembled those of the human brain during epilepsy that did 2D model (Chen et al., 2024). In this study, the 2D was unable to sustain long–term neural network activity and did not reproduce epilepsy signals similar to those observed in the human brain (Chen et al., 2024). The bioprinted model exhibited trends in the frequency, number of active channels, and amplitude of epilepsy signals that were much more similar to those observed in humans.

Richards et al. (2025) developed a bioprinted trophoblast organoid model using a synthetic PEG–based matrix to study the influence of the extracellular matrix on placental development and trophoblast differentiation. Proteomic comparison between conventional 2D cultures and bioprinted trophoblast organoids revealed enrichment of trophoblast stem cell markers (ISG15, SCD, SNX2), the cytotrophoblast marker CDH1, the proliferation marker Ki67, and extravillous trophoblast markers HLA–G, ITGA5, and QSOX1, indicating an enhanced trophoblast differentiation phenotype that is more representative of early placental tissue. Meng et al. (2019) developed a vascularized bioprinted lung cancer model incorporating tumor cells, fibroblasts, endothelial cells, and programmable growth factor gradients that recapitulated the key features of the tumor microenvironment. A direct comparison of the response of A549 cells to the immunotoxin EGF4KDEL in 2D and 3D cultures showed that the bioprinted model exhibited reduced sensitivity to EGF4KDEL treatment, highlighting the influence of cell–cell interactions, interacting among multiple cell types, and extracellular matrix components on therapeutic responses. Pichler et al. (2022) showed that bioprinted renal tubules induce transcriptional programs distinct from those in traditional 2D cultures. Immunofluorescence analyses demonstrated increased expression of kidney–associated markers, including ARG2 (arginase 2), a mitochondrial enzyme highly expressed in renal proximal tubules and involved in amino acid metabolism; NQO1 (NAD(P)H quinone dehydrogenase 1), a cytoprotective enzyme associated with oxidative stress responses; and OLFM1 (olfactomedin 1), a marker linked to epithelial differentiation and renal tissue organization. In addition, the bioprinted environment promoted the formation of organized tubular structures that closely resembled native kidney tissue architecture. Transcriptomic analyses revealed that 3D culture substantially altered gene expression patterns, with bioprinted tubules displaying enrichment in pathways associated with renal epithelial differentiation and tissue–specific function.

## 7 Comparison of spheroids, organoids, and bioprinting

Despite their structural and methodological differences, spheroids, organoids, and bioprinted tissues share several biological features that distinguish them from conventional 2D cultures, including three–dimensional architecture, cell–cell and cell–matrix interactions, diffusion gradients, and altered mechanotransduction (Forbes et al., 2014; Langhans, 2018; Clevers, 2016).

A challenge in comparing 2D and advanced 3D models is the lack of standardized, normalized, and well-reported strategies (Deben et al., 2024; Zhao et al., 2022). Depending on the study design and endpoint, results may be normalized to cell number, DNA content, protein concentration, tissue size, culture volume, or metabolic activity, limiting direct comparison across studies (Deben et al., 2024). This issue is particularly relevant for spheroids, organoids, and bioprinted tissues, where variations in size, cellular composition, extracellular matrix content, and growth kinetics can substantially affect assay outcomes (Zhao et al., 2022). Moreover, many analytical methods for emerging 3D platforms are still being optimized, underscoring the need for standardized quality–control metrics, normalization approaches, and reporting guidelines to improve reproducibility and cross–study comparability (Zhao et al., 2022; Lee et al., 2024).

Table 1 presents data related to their reproducibility, microenvironmental customization, vascularization potential, scalability, and the capacity to mimic native tissue complexity. Evidence from recent studies shows that 2D cultures offer high reproducibility and throughput, often becoming ready for experimentation within 24 h, but lack physiological relevance, limiting their predictive value for drug discovery and disease modeling (Garnique et al., 2024; Centeno et al., 2018; Sakolish et al., 2018). In contrast, spheroids generally require 24–72 h for formation and 1 week or more for maturation, improve cell–cell interactions, and enable more realistic gradients, although variability in size and structure remains a challenge despite advances in microfabrication and microfluidics. While the incorporation of endothelial, stromal, and immune cells can enhance spheroid complexity and functionality, these models still lack a functional vascular network and cannot fully reproduce the dynamic immune microenvironment present *in vivo* (Sarkar et al., 2018; Refet-Mollof et al., 2021; Moshksayan et al., 2018; Dornhof et al., 2022).

**TABLE 1:** Comparison of the key characteristics of spheroids, organoids, and Bioprinted *in vitro* models.

Characteristics	2D culture	Spheroids	Organoids	Bioprinting
Structural Control	Low, limited by flat substrate (Huang M. S. et al., 2025)	Moderate via self aggregation and microfabrication (Ahn et al., 2023)	Moderate, dependent on self organization (Peng et al., 2018)	High, with precise spatial control (Hu et al., 2025)
Reproducibility	High, standardized protocols (Jiang et al., 2020)	Moderate, size variability (Ahn et al., 2023)	Low–moderate, high batch variability (Peng et al., 2018)	Moderate–high, depends on parameter optimization (Kim et al., 2024)
Customization	Low (Huang M. S. et al., 2025)	Moderate via microenvironment tuning (Lu et al., 2025)	High, allows tissue specific identity (Ramesh and Palaniappan, 2025; Ye et al., 2020)	Very high due to bioinks and multi material printing (Hu et al., 2025; Wang et al., 2024)
Vascularization	Absent (Huang M. S. et al., 2025)	Weak, limited endothelial self organization (Lu et al., 2025)	Weak–emerging (Peng et al., 2018)	Best potential, including printed vascular networks (Chae et al., 2023)
Scale and Throughput	Very high (Shrestha et al., 2021)	Moderate–high with microfluidics (Wu et al., 2008)	Low–moderate, hard to scale (Jiang et al., 2020)	Moderate–high with high speed printing (Kim et al., 2024; Shin J. et al., 2025)
Mimicking Tissue Complexity	Low (Huang M. S. et al., 2025)	Moderate (Kaźmierski and Maj, 2023)	High, close to native tissue (Thorel et al., 2024)	Very high, enables spatial heterogeneity (Hu et al., 2025)
Co culture Potential	Limited; simple co cultures without architecture	Moderate; supports endothelial/stromal co cultures to improve gradients (Lu et al., 2025; Wu et al., 2008)	High intrinsic multicellularity but often needs engineered support for immune/vascular cells (Peng et al., 2018; Zhao et al., 2025)	Very high; precise spatial positioning of multiple cell types and ECMs (Huang M. S. et al., 2025; Hu et al., 2025; Chae et al., 2023)
Main Applications	Drug screening, basic research (Huang M. S. et al., 2025)	Drug screening, toxicology (Kaźmierski and Maj, 2023)	Disease modeling, personalized medicine (Thorel et al., 2024)	Tissue engineering, complex models (Kim et al., 2023)
Main Advantage	Simplicity and low cost	More realistic 3D cell–cell contacts (Lu et al., 2025)	High physiological fidelity (Thorel et al., 2024)	Precision and customization (Hu et al., 2025)
Main Limitation	Low physiological relevance (Huang M. S. et al., 2025)	Variability and limited structural complexity (Ahn et al., 2023)	Low reproducibility and poor vascularization (Peng et al., 2018)	Cost, complexity, and lack of standardization (Kim et al., 2023)

Compared with spheroids, organoids provide more biological fidelity to organs or tissues, mimicking native tissue organization and function, but require several weeks (typically 2–8 weeks) to achieve tissue–specific maturation and cellular complexity. However, despite these advances, most organoid systems still lack a functional vascular network and a fully integrated immune microenvironment, present batch variability, and are difficult to scale for high-throughput studies (Harrison et al., 2023; Czerniecki et al., 2018).

Bioprinting offers architectural precision and customization, facilitating the spatial arrangement of multiple cell types and biomaterials and supporting vascular constructs (Lawlor et al., 2021; Beheshtizadeh et al., 2020). However, these advantages come with challenges, as bioprinting workflows are often technically complex, resource**-**intensive, and require extensive optimization (Brauer et al., 2026; Zhu et al., 2016).

Moreover, the translational value of advanced 3D models is influenced by a range of experimental variables. Factors such as culture duration, extracellular matrix composition, cell source and donor–to–donor variability, spheroid size control, organoid maturation stage, bioprinting resolution, and reproducibility across independent laboratories can substantially affect the biological outcomes and assay performance. Therefore, these parameters should be carefully considered when interpreting the results and selecting an appropriate model, as they directly impact the reliability, predictive capacity, and diagnostic relevance of the generated data.

## 8 Applications of 3D models

Model selection should be guided by the intended application, as no single platform is optimal for all research goals. Instead, each model offers distinct advantages and limitations that should be considered in the context of the biological questions being addressed. Table 2 summarizes the application–based comparison of 2D, spheroid, organoid, and bioprinted models.

**TABLE 2:** Application–based comparison of 2D, spheroid, organoid, and bioprinted *in vitro* models.

Application/Criterion	2D	Spheroids	Organoids	Bioprinting
High throughput drug screening (HTS)	Best option; scalable and reproducible	Medium (batch variability)	Low–medium (complex, costly)	Medium (microarray bioprinting enables HTS)
Early regulatory toxicology	Recommended for initial exploratory testing	Better physiological relevance	Excellent human predictive value	Promising but high cost
Advanced drug toxicity/organ specific toxicity	Weak	Moderate	Strong organ specific predictivity	Strong for liver, cardiac, complex tissues
Spatial interactions/gradients/TME	Inadequate	Good	Excellent	Excellent (full spatial control)
Tissue development/organogenesis	Very limited	Limited	Best biological fidelity	Best structural engineering
Personalized medicine	Weak	Medium (patient derived spheroids)	Excellent (patient derived organoids)	Excellent for personalized constructs
Tissue engineering	Not recommended	Useful as microtissue building blocks	Good	Best option (geometry + ECM + vascularization)

## 9 NAMS future regulatory directions

Across research and development teams in industry and scientific laboratories in academia, spheroids, organoids, and other advanced 3D cellular models now serve as versatile workhorses for target discovery, disease modeling, patient stratification, and preclinical pharmacology or toxicology, with applications spanning oncology (tumor spheroid drug response), gastroenterology (intestinal barrier and microbiome co–culture), neurology (brain organoids for neurotoxicity), and nephrology (kidney organoids for nephrotoxicity screening and disease modeling), to name a few.

To advance from promising research tools to regulatory–grade methods, organoids/spheroids must clear validation milestones that are well recognized across regulatory agencies: 1) define a precise context of use (COU) aligned with a decision point; 2) analytical validation (i.e., accuracy, precision, dynamic range, limits of detection/quantitation, system suitability, and positive/negative control performance); 3) reliability and reproducibility, demonstrated within the laboratory and across independent sites; 4) biological relevance, benchmarked to human clinical data or high–confidence human reference sets; and 5) readiness for routine use, including standard operating procedure standardization (SOP), acceptance criteria, lot–to–lot matrix and cell–source controls, and data reporting packages.

In the regulatory pipeline, organoids/spheroids are being integrated into NAM strategies that the FDA is actively encouraging and building infrastructure to qualify via the Innovative Science and Technology Approaches for New Drugs (ISTAND) Drug Development Tool pathway. The ISTAND program is designed to grant context–of–use (COU) acceptance for tools that do not fit existing qualification programs. Although a variety of academic- and industry-based letters of intent utilizing organoids (e.g., an iPSC–cardiomyocyte Microelectrode Array (MEA) assay, InSphero’s human liver microtissue platform, MetaHeps, the Retrogenix platform, and the 3DInsight™ NASH assay) for specific COUs have been submitted to ISTAND, very few have progressed to the next phase of qualification plan development. Some examples of letters of intent using organoid platforms include: 1) a human iPSC-cardiomyocyte MEA assay that has been accepted for predicting clinical cardiovascular repolarization risk to support IND-enabling cardiac safety assessments, 2) a primary human liver spheroid model for preclinical drug-induced liver injury (DILI) risk assessment (not accepted), 3) the MetaHeps *ex vivo* platform for assessing drug causality in suspected immune-mediated DILI during clinical trials (under FDA review), 4) the Retrogenix specificity and off-target screening platform that has been accepted for enhancing biotherapeutic safety assessment across diverse antibody formats; and 5) a 3D human nonalcoholic steatohepatitis (NASH) model that has been proposed, but not accepted, for high-throughput screening and prioritization of NASH clinical candidates.

Letters of intent are often rejected because the submitted COU centers on preclinical tool development rather than explaining how the tool supports regulatory decision-making. Although the FDA acknowledges that developing preclinical screening tools is valuable for internal go/no-go decisions early in drug discovery, defining this as the COU for an organoid platform does not align with regulatory decision-making and falls outside the FDA’s regulatory authority.

Bioprinted tissues are emerging as promising components of NAMs and have been explored for drug screening, toxicology, disease modeling, and safety assessment because of their ability to recreate human–specific tissue architecture. However, unlike spheroids and organoids, bioprinted constructs frequently combine living cells, biomaterials, and additive manufacturing processes, creating regulatory challenges because they do not fit neatly within the traditional drug, biologic, or medical device categories. Consequently, regulatory oversight often necessitates case–by–case classification within frameworks such as Advanced Therapy Medicinal Products (ATMPs), Medical Device Regulations (MDRs), or combination–product pathways. Although regulatory agencies recognize the potential of bioprinting, wider regulatory acceptance depends on standardized manufacturing, quality control, reproducibility, analytical validation, and a clearly defined context of use (Perin et al., 2026).

These publicly available examples underscore the importance of identifying COUs that address specific needs within the regulatory pipeline. These examples highlight a promising future direction for spheroid, organoid, and bioprinted research, namely, to incorporate these models as reproducible and validated NAMs into weight–of–evidence frameworks to help shape regulatory decisions. Additionally, these models are primed to fit a distinct niche by bridging 2D assays and complex microphysiological systems across academic discovery, translational research, regulatory agencies, and industry sectors.

## 10 Future perspectives

Significant challenges remain in the development of highly physiologically relevant 3D *in vitro* models. For the current models under development, reproducibility across laboratories requires well–established protocol standardization. This includes not only technical procedures, but also complete documentation of the cell types used, including donor sex, ethnicity, and age. Additionally, a comprehensive analysis of variability across a large donor pool is required for a more–in–depth understanding. Further advances in multi–technology platforms are required to develop increasingly complex and physiologically relevant 3D models. A comprehensive analytical evaluation of 3D models requires advanced imaging and computational tools. In most cases, machine learning and artificial intelligence are crucial for elucidating the complex interactions within multimodal 3D systems.

These *in vitro* models, including 2D cultures, spheroids, organoids, and bioprinted tissues, are likely to converge into complementary, layered platforms designed to overcome the limitations of individual systems while accelerating regulatory acceptance, if advances in standardization, validation, and benchmarking against human reference tissues and *in vivo* data continue to be achieved (Wang et al., 2026; Zhou et al., 2025). Although 2D cultures remain indispensable for high–throughput screening because of their low cost, reproducibility, and scalability, they lack the structural and functional complexity necessary to accurately recapitulate native tissue physiology and predict human responses (Ingber, 2022; Jensen and Teng, 2020). Spheroids partially overcome these limitations by enhancing cell–cell interactions and establishing physiologically relevant gradients; however, variability in size, morphology, and maturation can still affect reproducibility and standardization (Bell et al., 2016; Tung et al., 2011). Organoids provide greater biological fidelity by reproducing key aspects of tissue architecture and function.

Bioprinting overcomes several structural limitations of current 3D models by enabling precise spatial organization, defined tissue geometry, controlled perfusion, and more standardized tissue manufacturing. Nevertheless, its broader translation will depend on continued improvements in bioink development, biocompatibility, printing resolution, scalability, the establishment of harmonized regulatory guidelines, and manufacturing costs (Huang M. S. et al., 2025; Srinivasan et al., 2020; Li et al., 2025; Le et al., 2025).

However, several barriers remain. The implementation of an automated workflow, including robotic platforms and artificial intelligence, has been shown to be effective in reducing, at least in part, the variability associated with human–induced errors (Renner et al., 2021). In this context, the use of well–defined SOPs is essential for improving intralaboratory reproducibility in environments that lack automatic and AI assistance, thereby reducing phenotypic heterogeneity among organoid batches. However, the scalability, cost, and limited accessibility of bioprinting technologies restrict their broader implementation despite their substantial promise.

Future progress will likely rely on the development of integrated platforms that combine the strengths of spheroids, organoids, bioprinting, and microfluidic systems. Future innovations, including advanced biomimetic surfaces, multi–omics integration, and artificial intelligence–assisted analysis, are expected to improve their physiological relevance and predictive performance while preserving their scalability and throughput. Spheroid technology is advancing through automated generation platforms employing microfluidic devices, acoustic levitation, and magnetic bioprinting to achieve a uniform size distribution and composition. Organoid technology is undergoing rapid standardization through defined culture conditions, synthetic matrices that replace animal–derived Matrigel, and automated culture systems that minimize operator–dependent variability. Micropatterning and bioengineering approaches have been developed to control organoid size, shape, and cellular composition, thereby enhancing their reproducibility for drug screening applications. Bioprinting is advancing through next–generation bioinks that balance printability, biocompatibility, and mechanical properties while supporting long–term cell viability and function, including cell–laden hydrogels incorporating growth factors, extracellular matrix components, and stimuli–responsive materials that enable dynamic remodeling. High–resolution printing techniques, such as two–photon polymerization and volumetric bioprinting, enable the construction of capillary networks and complex tissue interfaces. Ultimately, the convergence of these technologies may enable the development of increasingly predictive human–relevant models for disease research, drug development, precision medicine, and improved preclinical safety.

Rather than replacing one another, 2D cultures, spheroids, organoids, and bioprinted tissues are expected to become complementary components of increasingly predictive human–based experimental platforms that will accelerate biomedical research and reduce reliance on animal models.

## 11 Conclusion

Spheroids, organoids, and bioprinted models have demonstrated enhanced physiological relevance compared with conventional 2D cell cultures in various aspects, including a more adequate recapitulation of the 3D architecture of native tissue, cellular polarity, nutrient gradients, cell–extracellular matrix interactions, and long–term culture capabilities. These aspects have generated systems that align more closely with native tissues, including *in vivo*–like tissue architecture, drug sensitivity, differential gene expression, increased expression of tissue–specific markers, and increased or more stable metabolic and transporter functions over time. In many cases, vascular or pro–angiogenic signatures and ECM maturation are present. Finally, they can differ from 2D in terms of toxicological sensitivity, which is mainly context–and compound–dependent. Accordingly, advanced 3D models are emerging as key components of the expanding NAM toolbox, supporting the transition toward more human–relevant and animal–free approaches in biomedical research.
