# Supplementary material for: Beyond monolayers: a comparative analysis of 2D cell cultures and 3D in vitro models as new approach methodologies
Source: Front Bioeng Biotechnol. 2026 Aug 24;14:1862040. doi: 10.3389/fbioe.2026.1862040 (PMC13548193; doi:10.3389/fbioe.2026.1862040)
Supplement: Supplementary file 1 [file Table1.docx]

Supplementary Information

**Beyond Monolayers: A comparative analysis of 2D cultures and 3D *in vitro* models as new approach methodologies**

Authors: Italo Rodrigo Calori^1a^, Ana Paula Pereira Guimaraes^1a^, Ananth Kumar Kammala^1^, Lauren Richardson^1^, Ramkumar Menon^1*^

^1^Department of Obstetrics & Gynecology, Division of Basic Science and Translational Research, The University of Texas Medical Branch at Galveston, Galveston, TX, USA

^a^These authors contributed equally to this work

^*^Corresponding author:

Ramkumar Menon

Professor & Vice Chair for Research

Department of Obstetrics and Gynecology

Director, Translational Research Center (TraCe) Microphysiologic System (MPS) (NIH/NCATS)

The University of Texas Medical Branch at Galveston

MRB 11-138, 301 University Blvd, Galveston, TX 77555

Ph (Office) - 409 -772-7596; Ph (Cell) - 615-335-5564

Keywords: spheroid, organoid, bioprinting, 3D *in vitro* model, new approach methodologies.

| **Table S1.** Differences observed in 3D spheroid models compared with 2D culture | | | | | | | |
| --- | --- | --- | --- | --- | --- | --- | --- |
| Tissue model | Cell lines | Gene/protein expression | Secretion | Morphology | Drug response | Others | Ref. |
| Adposite | Primary Human Subcutaneous SVF Cells | ↑ differentiation-dependent genes (LIPE, FABP4, ADIPOQ) | ↑ adiponectin and leptin | ↑ unilocular morphology | - | - | [1] |
|  | MSCs |  | ↑ adiponectin | - | - | Facilitated differentiation of lipid-rich adipocytes | [2] |
|  | Adipose-Derived Stem Cells (ASCs) and mature adipocytes (ACs) | No significant increase in adipocyte-specific genes during maintenance | - | rounder cells | - | ↑ adipogenic potential / facilitated differentiate and accumulate intracellular lipids | [3] |
|  | 3T3-L1 mouse preadipocytes | ↑ Key adipogenic genes like glucose transporter 4 (Glut4), fatty acid transporters (FATs), fatty acid binding proteins (FABP), perilipin, PPAR-α, and PPAR-γ | - | - | Linoleic acid (LA) and TNF-α | ↑ intracellular triglyceride accumulation; | [4] |
|  |  | ↓ matrix metalloproteinases (MMPs), integrin, and actin-cytoskeleton associated genes, as well as Rho/GTPase3 expression |  |  |  | ↑sensitivity to fatty acid and inflammatory stimuli, such as TNF-α | [4] |
| Brain | HASTR/ci35 | ↑ astrocytic differentiation markers (glutamine synthetase (GS), excitatory amino acid transporter 2 (EAAT2), glial fibrillary acidic protein (GFAP), and aldehyde dehydrogenase 1 family member L1 (ALDH1L1)), ↑ inflammatory response genes (PTGS2, CXCL8, CCL5, LCN2) | Laminin deposition | - |  | ↑ cell-cell interactions and extracellular matrix accumulation | [5] |
|  | SH-SY5Y human neuroblastoma cell line | - | - | - | 2D model showed higher sensitivity than the 3D model for both exposure times (24h and 96h), such as cell viability, area change, ROS, and cell cycle phases |  | [6] |
|  | Stem-cell-derived neuronal spheroids (3D-hNLCs) | - | - | specific architecture, cell morphology, and cell-to-cell and cell-to-extracellular matrix (ECM) interactions | Methylglyoxal (MGO) inducesd neurotoxic | - | [7] |
| Female Reproductive System | Immortalized human uterine leiomyoma (ht-UtLM) | - | - | - | PD98059, SB203580, and SP600125 as inhibitors in the upstream analysis of TGF-beta signaling pathways | ↑ collagen deposition | [8] |
| Heart | Cells derived from mouse cardiac explants | ↑ vascular progenitor cell markers (Pdgfra, Kit, and Vegfr1) and angiogenesis regulators (Vegf, Fgf2, and Angpt1) | - | - |  | - | [9] |
|  | Human cardiomyocytes derived from induced-pluripotent stem cells (hiPSC-CMs) | 3D model showed more tissue-like expression pattern of selected cytoskeleton and myofibrillar proteins | - | cardiac spheroids lose stress fibers and alpha-SMA expression, reverting to a phenotype similar to normal cardiac tissue | Doxorubicin (Doxo) 3D models were found to be more resistant to anticancer drugs |  | [10] |
| Kidney | Human adult renal progenitor cells (ARPCs) and CD133-cells | Greater expression of stem cell markers (SOX2, OCT3/4, GATA3) | ↑ renin | - |  | (3D) induced a stronger angiogenic response | [11] |
|  | HK-2 cells | ↑ expression of kidney development-related genes, such as WNT9B | - | - | Ouabain octahydrate - 3D culture cells exhibited higher Na retention | induces apical-basal polarity, restores Na transport function | [12] |
|  | RPTECs (Renal Proximal Tubular Epithelial Cells) and HK-2 | Expression levels of drug transporters (OAT1, OCT2, OAT3, URAT1, MATE1, MATE2K, megalin, P-gp, MRP2, and MRP4) and intracellular ATP levels for up to 28 days, | - | - | ATP levels was reduced by Cisplatin and tenofovir in 3D-RPTECs but remained unchanged in 2D | ↑ sensitivity and specificity for DIKI | [13] |
|  | Conditionally immortalised (CI) human podocytes and glomerular endothelial cells (GEnCs) | ↑ key podocyte markers (podocin, nephrin, podocalyxin) and GEnC marker (pecam-1) | - | podocyte foot process interdigitation and endothelial vessel formation. | Nintedanib demonstrated anti-fibrotic properties | mature, organized isoforms of collagen IV and Laminin, with mature collagen IV a3 and laminin a5 being significantly higher | [14] |
|  | Mouse tubular cell line (TCMK-1), hPTEC | ↑ expression of major uptake and efflux drug transporters | - | - | ↑ sensitivity to nephrotoxic compounds (cisplatin and CsA), sensitive apoptosis profiles and specific transporter activity responses to nephrotoxicants |  | [15] |
| Liver | HepG2 and HepaRG | Gene expression of xenobiotic-metabolizing enzymes, as well as increased CYP activities | ↑ albumin | - | Phenacetin, Bupropion (CYP2B6: ↓ activities in 2D), Amodiaquine, Tolbutamide (CYP2C9: ↑ activity in 2D), S-mephenytoin (CYP2C19: ↑ levels in 3D), Propafenone and midazolam (CYP3A4:↑ levels in 3D) . | - | [16] |
|  | HepG2 | - | - | - | 3D ↑ toxity and sensitivity to acetaminophen, diclofenac, fialuridine, trovafloxacin |  | [17] |
|  | HepG2 | ↑ induction CYP3A4 and CYP1A2 | ↑ liver-functions (albumin and urea secretion) | - | 3D ↑ sensitivity to acetaminophen |  | [18] |
|  | HepaRG | ↑ CYP activity in 3D spheroids | - | - | ↑ genotoxic and cytotoxic (nitrosamine drug ) | ↑ metabolic activity | [19] |
|  |  |  |  |  |  | 3D more closely mimimicking *in vivo* | [19] |
|  | HepaRG | ↑ CYP activity levels | - | - | ↑ induced DNA damage, cytotoxicity and genotoxicity ( nitrosamine drug ) |  | [20] |
|  | Neuroblastoma SH-SY5Y, epithelial breast cancer MDA-MB-213, bone marrow-derived mesenchymal stem cells (BM-MSC) and umbilical vein endothelial cells (HUVECs) | - | - | - | ↑ resistance in 3D model ( sterigmatocystin, ochratoxin A, and patulin) |  | [21] |
|  | Primary human hepatocytes (PHHs) | ↑ CYP activities (CYP3A4, CYP2C9, CYP1A2) | Albumin and urea drug, and bile transporters, | - | Acetaminophen (APAP), amiodarone (AD), bosentan (BST), cyclosporin A (CsA), and diclofenac (DCLF). | - | [22] |
|  | McA-RH7777 cells, an immortalized rat hepatoma cell line | ↑ gene expression related to xenobiotic metabolism (AhR and PPAR) after treatment | - | - | ↑ sensitivity and uniform dose-response; ↑ Sgk1, Cd36, Cyp1a1, Angptl4, Cd36, Pgc1a after treatment with Dexamethasone |  | [23] |
|  |  |  |  |  | ↑ Sgk1, Cd36, Cyp1a1, Angptl4, Cd36, Pgc1a after treatment with Dexamethasone |  | [23] |
|  | HepaRG | ↑ express liver-specific markers and drug-metabolizing enzymes | - | - | ↑ mitochondrial membrane potential , lipid levels in 3D model ( Clofibrate (CF) ) | ↑ similarity to complex tissue-like architecture, | [24] |
|  | HepG2, PUMC-HUVEC-T1, THP-1 derived macrophages, and HHSteCs | ↑ drug metabolic enzyme expression and CYP3A4 activity | ↑ albumin and urea | - | ↑ sensitivity to compound cytotoxicity (22 DILI-positive and 5 DILI-negative) |  | [25] |
|  | Fish liver cell line RTL-W1 | ↑ ahr, cypla, fatp1 and abcc2 |  | - | surfactant SDS and the metal copper (Cu) | tissue-like arrangements with cell-cell junctions and ultrastructural characteristics typical of metabolically active cells | [26] |
| Lung | Primary human lung fibroblasts (HLFs) | ↑ stemness markers (OCT4, SOX2, NANOG) | - | - | ↑ sensitivity for PM2.5 toxicity analysis |  | [27] |
| Ovary | CaOV3, OVCAR3, OVCAR4, OVCAR5, OVCAR8, COV318, COV362, HeyA8, iOvCa147-MA and immortalized human fallopian tube secretory epithelial cell line FT190 | ↑ LC3-II processing and phosphorylated AMPK | - | - | Compound C |  | [28] |
| Pancreas | Human pluripotent stem cells (hPSC) | ↑ expression Sox17, FOXA2, HNF4a, HNF1b, NGN3 mRNA, insuline, β cell markers such as PDX1, NKX6.1, and MAFA.↑ glucose-sensing apparatus marker genes (GCK, KIR6.2, SUR1, Glut2) | ↑ insuline | - | Glucose, potassium chloride, and Forskolin sensitivity |  | [29] |
|  |  | ↑ glucose-sensing apparatus marker genes (GCK, KIR6.2, SUR1, Glut2) |  |  |  |  | [29] |
|  | MIN6 cells (mouse islet β cell line) | ↑ expression of islet secretion-related genes (GLUT2, PDX1, INS2); ↑ PI3K, p-AKT/AKT protein expression | Insuline | - | PI3K inhibitor reduced GSIS reaction |  | [30] |
|  |  | ↑ PI3K, p-AKT/AKT protein expression |  |  |  |  | [30] |
|  | hiPSC derived β-cells | ↑ gene expression of PDX1, NKX2.2, NKX6.1, and INS; ↓ mRNA GCK, UCN3, NGN3; ↑ mRNA GCG, SST, and GLUT2 | Positive C-peptide secretion | - | GLP1 (Glucagon-Like Peptide 1) | Spheroids showed positivity for glucagon | [31] |
|  |  | ↓ mRNA GCK, UCN3, NGN3 |  |  |  |  | [31] |
|  |  | ↑ mRNA GCG, SST, and GLUT2 |  |  |  |  | [31] |
| Placenta | HTR8/SVneo EVT | ↑ canonical pathways and biological processes: immune response, angiogenesis, response to stimulus, and wound healing; ↑ EMT markers (WNT5A, TGFB1, TGFB2, ITGA2, ITGA5, ITGB1, VIM); ↑ cell-cell contact/tight junction markers (CDH1, TJP1, CLDN1, CLDN4); ↑ angiogenesis and invasion markers (HIF1A, VEGFA, VEGFC, MMP9). | - | - | more predictive response and resistance to drug exposure |  | [32] |
|  |  | ↑ EMT markers (WNT5A, TGFB1, TGFB2, ITGA2, ITGA5, ITGB1, VIM); |  |  |  |  | [32] |
|  |  | ↑ cell-cell contact/tight junction markers (CDH1, TJP1, CLDN1, CLDN4) |  |  |  |  | [32] |
|  |  | ↑ angiogenesis and invasion markers (HIF1A, VEGFA, VEGFC, MMP9) |  |  |  |  | [32] |
|  | BeWo | ↑ syncytialization-related genes (GCM1, PL, ERVWE1, CGB); ↑ ITGA1 and ITGA5 mRNA; ↑ MMP2 and TIMP1 mRNA | ↑ human chorionic gonadotropin β (hCGβ) | - |  |  | [33] |
|  |  | ↑ ITGA1 and ITGA5 mRNA |  |  |  |  | [33] |
|  |  | ↑ MMP2 and TIMP1 mRNA |  |  |  |  | [33] |
|  | BeWo, JEG-3, and HTR-8/SVneo | Gene expression similarities with primary placental tissue | ↑ β-HCG ( BeWo and JEG-3 3D culture) | - |  |  | [34] |
|  | Placenta-derived mesenchymal stem cells (MSCs) |  | - | spheroid culture significantly reduces cell size and enhances immunomodulatory function | Dexamethasone (Dex) | ↑ levels of IL-10; | [35, 36] |

**Abbreviations:** AhR, aryl hydrocarbon receptor; AMPK, AMP-activated protein kinase; Angpt1, angiopoietin-1; BM-MSC, bone marrow-derived mesenchymal stem cell; CI, conditionally immortalized; CYP, cytochrome P450; DIKI, drug-induced kidney injury; DILI, drug-induced liver injury; EAAT2, excitatory amino acid transporter 2; ECM, extracellular matrix; EMT, epithelial-to-mesenchymal transition; FABP, fatty acid-binding protein; FATs, fatty acid transporters; Fgf2, fibroblast growth factor 2; GFAP, glial fibrillary acidic protein; GEnCs, glomerular endothelial cells; GLP1, glucagon-like peptide 1; GS, glutamine synthetase; GSIS, glucose-stimulated insulin secretion; hPSC, human pluripotent stem cell; HIF1A, hypoxia-inducible factor 1 alpha; IL-10, interleukin 10; LC3-II, microtubule-associated protein 1 light chain 3-II; MGO, methylglyoxal; MMPs, matrix metalloproteinases; OAT1, organic anion transporter 1; OAT3, organic anion transporter 3; OCT2, organic cation transporter 2; PHHs, primary human hepatocytes; PI3K, phosphoinositide 3-kinase; PM2.5, particulate matter ≤ 2.5 μm; ROS, reactive oxygen species; SGK1, serum/glucocorticoid regulated kinase 1. **Symbols:** ↑ = increased, enhanced, or upregulated; ↓ = decreased, reduced, or downregulated.

**Table S2.** Differences observed in 3D organoids models compared with 2D culture

| Tissue model | Cell lines | Gene/protein expression | Secretion | Morphology | Drug response | Others | Ref. |
| --- | --- | --- | --- | --- | --- | --- | --- |
| Placenta | Cryopreserved term cytotrophoblast isolates |  | ↑ hCG |  |  | spontaneously expressed β-hCG; self-organise into a multinucleated syncytiotrophoblast inner-layer | [37] |
|  | TSC lines (BTS5, BTS11, CT27, CT29, and CT30) and Seven patient-derived TO (trophoblast organoids) lines |  | ↑ hCG-β |  |  | TSC 3D : ITGA2+ progenitor-like cells, EVT-biased profile with limited spontaneous SCT formation TO: villous/VCT progenitors, more physiologically relevant HLA-G+ HLA-C+ EVT | [38] |
| Pancreas | Pancreatic adipose tissue-derived stromal vascular fraction (SVF) cells | ↑mRNA levels of PPARG and adiponectin (ADIPOQ); ↑ Beta-adrenergic receptors (ADRB1, ADRB2, ADRB3) | ↑ FFA and glycerol |  |  | improved adipogenesis and functional characteristics similar to mature adipocytes /↑ adipogenesis and maintained a donor-related phenotype | [39] |
|  | Mouse pancreatic ductal cells |  |  |  |  | ductal characteristics, whereas those grown in 2D culture undergo an epithelial-mesenchymal transition (EMT) | [40] |
|  | Human PSCs | 3.2-fold increased differentiation efficacy; purified PDX1+ SOX17+ TBX3- molecular phenotype |  |  |  | self-organized ; epithelial and mesenchymal compartments | [41] |
| Lung | Human alveolar type 2 (hAT2) cells,Calu-3, NCI-H1299, and Caco-2 |  |  |  |  | more physiologically relevant system (viral replication, host immune responses, and cellular pathogenesis) | [42] |
|  | Broncho-alveolar resections |  |  |  |  | long-term expansion,  polarized, pseudostratified airway epithelium containing basal, secretory, and multi-ciliated cells | [43] |
|  | Bronchial tissue (BT), Bronchoalveolar lavage (BAL) and tracheal aspirate (TA) derived AEC |  |  |  | cigarrete smoke: ↑ HMOX1 and NQO1 expression after 3 h of treatment |  | [44] |
| Liver | Human iPSC-HEs, HUVECs, human MSCs and iPSC-LBs | ↑ FGF and BMP pathways | ↑ albumin | self-organized into macroscopically visible three-dimensional cell clusters in organoid culture, | Ketoprofen and debrisoquine |  | [45] |
|  | Human pluripotent stem cells (PSCs), human embryonic stem cells (hESCs) and induced pluripotent stem cells (iPSCs) | ↑ pluripotency marker NANOG; maintained expression of the adult stem cell marker; ↑ CYP3A7 ; ↑SOX9 and CK19 (ductal markers) and MH markers ALB, TTR, CK18, and RBP4 (mature hepatocutes markers); ↑ levels CYP families | ↑ ATT, ↑ ALB | Polarized epithelial cells with bile canaliculi-like structures |  | liver similarity scores / ↑ ATP production | [46] |
|  | Human pluripotent stem cells (hPSCs) and hepatocyte-like cells (2D HLCs) | ↑ CYP2B6, CYP2C9, CYP2C19, and CYP3A4 gene expression; ↑ drug transporter genes (MDR1 and MRP2); ↑ H3K27ac mark |  | nduction of CYP3A4, UGT1A1, and MDR1, mediated by constitutive androstane receptor (CAR) and pregnane X receptor (PXR) |  | ↓ DNA methylation and ↑ histone acetylation / mature properties | [47] |
|  | PHHs (primary human hepatocytes) and HuCCT-1 cells (human bile duct carcinoma cell line) |  |  |  |  | ↑ differentiation and functionality/ lumen-like | [48] |
| Kidney | WA09 (H9) huma human pluripotent stem cells (hPSCs)n ESCs and WTC11 human iPSCs | KIM-1 expression |  |  | Cisplatin and Blebbistatin | Single-cell RNA-seq -> human kidney complexity; cyst formation | [49] |
| Gut | Human pluripotent stem cells (hPSCs), intestinal epithelial cell (hIEC) |  |  |  | PM10 | mimic *in vivo* cellular diversity and function, displaying various intestinal cell types and specific functionality | [50] |
| Heart | Human pluripotent stem cells (hPSCs), iPSC-derived cardiac differentiating cells | ↑ heart-specific extracellular matrix genes; progile closer to fetal hearts / ↑ expression of genes associated with conduction, contractile function, calcium handling, and various cardiac cell populations |  |  |  | ↑ cardiac cell lineage complexity | [51] |
|  | HES3 NKX2.5-eGFP human embryonic stem cells (hESCs), hiPSC line HSC_ADCF_SeV-iPS2 | ↓ cardiac markers and ↑ endoderm markers |  |  |  | endodermal components | [52] |
|  | NKX2-5eGFP/W hESCs | ↓contractility, ↓ cardiac contractile and maturation-related gene expression, |  |  | higher dose effect in the 3D (13-cis-Retinoic acid) |  | [53] |
| Brain | Pluripotent stem cell (hiPSC) lines |  |  | heterogeneous cell populations |  | early stages of human cortical development; | [54] |
|  | cell lines mfNPC H1, H2, H3, and H4; mfNPC H1, P1-GC, and P2-GC |  |  | multiple region-specific cell types |  |  | [55] |
|  | Human iPSC-derived neural progenitor cells (NPCs) |  |  |  | more sensitive to ivermectin, palonosetron, and pyrimethamine in functional assay / lower senritive to mefloquine and sertraline |  | [56] |

**Abbreviations:** AAT, alpha-1 antitrypsin; AEC, airway epithelial cell; BAL, bronchoalveolar lavage; BMP, bone morphogenetic protein; BT, bronchial tissue; CAR, constitutive androstane receptor; EVT, extravillous trophoblast; FFA, free fatty acids; hAT2, human alveolar type 2 cell; hCG, human chorionic gonadotropin; HLA, human leukocyte antigen; HLCs, hepatocyte-like cells; iPSC-HEs, induced pluripotent stem cell-derived hepatic endoderm cells; iPSC-LBs, induced pluripotent stem cell-derived liver buds; KIM-1, kidney injury molecule 1; MDR1, multidrug resistance protein 1; MH, mature hepatocyte; MRP2, multidrug resistance-associated protein 2; NQO1, NAD(P)H quinone dehydrogenase 1; PM10, particulate matter ≤10 μm; PSCs, pluripotent stem cells; PXR, pregnane X receptor; RNA-seq, RNA sequencing; SCT, syncytiotrophoblast; SVF, stromal vascular fraction; TA, tracheal aspirate; TO, trophoblast organoid; TSC, trophoblast stem cell; UGT1A1, UDP glucuronosyltransferase family 1 member A1; VCT, villous cytotrophoblast. **Symbols:** ↑ = increased, enhanced, or upregulated; ↓ = decreased, reduced, or downregulated.

**Table S3.** Differences observed in 3D printing models compared with 2D culture

| Tissue model | Cell lines | | Gene/protein expression | Secretion | | | Morphology | | Drug response | | Others | | Ref. |
| --- | --- | --- | --- | --- | --- | --- | --- | --- | --- | --- | --- | --- | --- |
| Lung cancer | H358 cell line | |  |  | | |  | | ↑ (1000-fold) gemcitabine concentrations needed to induce cell death | | Considered closer to the biological response in human patients | | [57] |
| Brain cancer | IMR-32 cells (neuroblastoma), HEK293 cells and primary human kidney fibroblasts | |  |  | | | Sensitivity of3D (HEK293 and IMR-32 cells) to panobinostat and blasticidin | |  | |  | | [58] |
| Breast cancer | MCF-7 cells (human breast cancer cells)  hAMSCs (human adipose-derived mesenchymal stem cells) | | ↑ Tumor Markers [COL1A1 (collagen type 1 alpha 1)]; ↑ HSP90AA1 and HSP90AB1 genes; ↓ FN1; ↑ Multidrug Resistance Proteins (ABCC1 and ABCG2) |  | | |  | | Doxorubicin sensitivity was significantly lower in 3D bioprinted cultures compared to 2D cultures | | CACNA1G and KCNA1 expression absence; more accurate representation of the tumor microenvironment in terms of gene expression and the absence of certain ion channels | | [59] |
| Brain cancer | Human primary astrocytes Pericytes Brain microvascular endothelial cells Patient-derived glioblastoma cells (JHH-520) | | ↑ Cytokine-related genes |  | | |  | |  | | Transcriptomic Changes GBM Subtypes and Gene expression; | | [60] |
| Brain | Induced pluripotent stem cell (iPSC)-derived astrocytes  Brain microvascular endothelial-like cells (BMECs)  Neural progenitors (NPCs)  Neurons | |  | no significant increase in IL-6 secretion upon LPS stimulation | | |  | |  | | ↑ the number of spontaneous calcium spikes per neuron | | [61] |
| Brain | Astrocyte, Primary neural cells | |  |  | | | 3D bioprinted constructs offer a spatial environment that better simulates mechanical properties for neural cells, allowing 3D growth and extensio | |  | | Lower epilepsy signals detection, but overall characteristics more complex, realistic and stable epilepsy signal | | [62] |
| Pancreas | NS-1 832/3 cell line and HUVEC | | ↑ hallmark pathways (e.g., pancreas B-cells, TGFβ signaling, angiogenesis); ↓ adipogenesis, reactive oxygen species pathway, oxidative phosphorylation) | insuline | | |  | |  | | Enrichment of pancreatic B-cell-specific genes; Physiological insulin response comparable to healthy human islets | | [63] |
| Kidney | ureretic bud iPSC | |  |  | | |  | |  | | Supported complex branching and luminal structures | | [64] |
|  | Immortalized renal proximal tubule epithelial cells (RPTEC) and HUVEC | | ↑ JAG1 |  | | |  | |  | | cystic aggregates; endothelial co-culture improved metabolic activity | | [65] |
|  | renal tubular epithelial cells (iRECs) | | Cdh1, Cdh16, Lrp2, and Slc17a1 being preserved or differentially regulated; 322 genes concordantly up- or downregulated; ↑ Nqo1, Arg2, Olfm1 |  | | |  | |  | | ↑ proliferation rates; | | [66] |
| Vascular | Human umbilical vein ECs (endothelial cells) and human umbilical artery smooth muscle cells (VSMCs) | |  |  | | |  | |  | | co-culture of ECs and VSMCs in a 3D cylindrical structure, mimicking the physical and chemical microenvironments of native human vasculature | | [67] |
| Skin cancer | A375 melanoma cells | |  |  | | | ↑ sensitivity to drug-induced apoptosis | |  | | more effectively replicate the *in vitro* microarchitecture of tumors | | [68] |
| Breast cancer | MCF7 and MDA-MB-231 breast cancer cell lines | |  |  | | | more resistant to cisplatin | |  | | ↑ reactive oxygen species levels;mimic tumor heterogeneity and microenvironment | | [69] |
| Brain cancer | SK-N-BE(2) neuroblastoma cells | | T Cells: ↑ CD25 and CD137 activation markers | ↓ levels of interferon gamma (IFNG) | | | strong L1CAM-specific CAR T cell activation | |  | | enables the analysis of CAR T cell infiltration and phenotyp | | [70] |
| Cancer | A549 lung cancer cells, M4A4 melanoma cells and HUVEC | |  |  | | |  | |  | | provide more meaningful insights into *in vivo* cellular response to drugs, including cell-cell and cell-ECM interactions, endothelial permeability, and stroma influence | | [71] |
| Brain cancer | Human U87-MG glioblastoma | |  |  | | | Ker-AuNPs did not further affect viability (87.7% for 3D + Ker-AuNPs vs. 85% for 3D) | |  | | lower toxicity and are significantly correlated with *in vivo* animal studies | | [72] |
| Brain cancer | Neuroblastoma IMR-32 cells  Human embryonic kidney 293 cells (HEK293)  ]Primary human kidney fibroblasts | |  |  | | | IC50 values of IMR-32 cells for panobinostat were approximately one order of magnitude higher for 3D cultures than for 2D monolayers; | |  | |  | | [58] |
| Placenta | | ACH-3P | Enhanced trophoblast-associated pathways; TNF-α reduced viability/metabolism. | |  |  | | No rescue by aspirin or metformin | | Greater physiological complexity and altered drug responses | | [73] | |

**Abbreviations:** ABCC1, ATP-binding cassette subfamily C member 1; ABCG2, ATP-binding cassette subfamily G member 2; BMECs, brain microvascular endothelial-like cells; CAR-T, chimeric antigen receptor T cells; ECs, endothelial cells; GBM, glioblastoma; HSP90AA1, heat shock protein 90 alpha family class A member 1; HSP90AB1, heat shock protein 90 alpha family class B member 1; IC50, half-maximal inhibitory concentration; IFNG, interferon gamma; IL-6, interleukin 6; JAG1, jagged canonical Notch ligand 1; Ker-AuNPs, keratin-coated gold nanoparticles; L1CAM, L1 cell adhesion molecule; LPS, lipopolysaccharide; TGFβ, transforming growth factor beta; VSMCs, vascular smooth muscle cells. **Symbols:** ↑ = increased, enhanced, or upregulated; ↓ = decreased, reduced, or downregulated.

**References**

[1] A. Ioannidou, S. Alatar, R. Schipper, F. Baganha, M. Åhlander, A. Hornell, R.M. Fisher, C.E. Hagberg, Hypertrophied human adipocyte spheroids as in vitro model of weight gain and adipose tissue dysfunction, The Journal of physiology, 600 (2022) 869-883.

[2] J. Liszewski, A. Klingelhutz, E.A. Sander, J. Ankrum, Development and analysis of scaffold-free adipose spheroids, Adipocyte, 13 (2024) 2347215.

[3] F.B. Albrecht, A.K. Schick, A. Klatt, F.F. Schmidt, S. Nellinger, P.J. Kluger, Exploring Morphological and Molecular Properties of Different Adipose Cell Models: Monolayer, Spheroids, Gellan Gum‐Based Hydrogels, and Explants, Macromolecular bioscience, 25 (2025) 2400320.

[4] P.A. Turner, M.R. Garrett, S.P. Didion, A.V. Janorkar, Spheroid culture system confers differentiated transcriptome profile and functional advantage to 3T3-L1 adipocytes, Annals of biomedical engineering, 46 (2018) 772-787.

[5] F. Tao, K. Kitamura, S. Hanada, K. Sugimoto, T. Furihata, N. Kojima, Rapid and stable formation method of human astrocyte spheroid in a high viscous Methylcellulose medium and its functional advantages, Bioengineering, 10 (2023) 349.

[6] M. Sendra, M. Cavia-Saiz, P. Múñiz, Are the BPA analogues an alternative to classical BPA? Comparison between 2D and alternative 3D in vitro neuron model to assess cytotoxic and genotoxic effects, Toxicology, 502 (2024) 153715.

[7] T. Coccini, F. Caloni, L.A. Russo, L. Villani, D. Lonati, U. De Simone, 3D human stem-cell-derived neuronal spheroids for in vitro neurotoxicity testing of methylglyoxal, highly reactive glycolysis byproduct and potent glycating agent, Current Research in Toxicology, 7 (2024) 100176.

[8] J. Liu, L. Yu, L. Castro, Y. Yan, P. Bushel, E. Scappini, D. Dixon, Induction of fibrosis following exposure to bisphenol A and its analogues in 3D human uterine leiomyoma cultures, Journal of hazardous materials, 476 (2024) 134772.

[9] Y.D. Vasilets, K. Dergilev, Z. Tsokolaeva, E. Parfenova, Culturing of cardiac cells in 3D spheroids modulates their expression profile and increases secretion of proangiogenic growth factors, Bulletin of Experimental Biology and Medicine, 173 (2022) 235-239.

[10] P. Beauchamp, C.B. Jackson, L.C. Ozhathil, I. Agarkova, C.L. Galindo, D.B. Sawyer, T.M. Suter, C. Zuppinger, 3D co-culture of hiPSC-derived cardiomyocytes with cardiac fibroblasts improves tissue-like features of cardiac spheroids, Frontiers in molecular biosciences, 7 (2020) 14.

[11] F. Giannuzzi, A. Picerno, S. Maiullari, F. Montenegro, A. Cicirelli, A. Stasi, G. De Palma, V.F. Di Lorenzo, G.B. Pertosa, P. Pontrelli, Unveiling spontaneous renal tubule-like structures from human adult renal progenitor cell spheroids derived from urine, Stem cells translational medicine, 14 (2025) szaf002.

[12] K. Mizuguchi, H. Aoki, M. Aoyama, Y. Kawaguchi, Y. Waguri-Nagaya, N. Ohte, K. Asai, Three-dimensional spheroid culture induces apical-basal polarity and the original characteristics of immortalized human renal proximal tubule epithelial cells, Experimental cell research, 404 (2021) 112630.

[13] H. Arakawa, D. Higuchi, E. Takahashi, K. Matsushita, S. Nedachi, H. Peng, M. Kadoguchi, K. Morimura, A. Araki, M. Kondo, Three-dimensional culture of human proximal tubular epithelial cells for an in vitro evaluation of drug-induced kidney injury, Journal of Pharmaceutical Sciences, 113 (2024) 3255-3264.

[14] J. Tuffin, M. Chesor, V. Kuzmuk, T. Johnson, S.C. Satchell, G.I. Welsh, M.A. Saleem, GlomSpheres as a 3D co-culture spheroid model of the kidney glomerulus for rapid drug-screening, Communications biology, 4 (2021) 1351.

[15] H.M. Kang, J.H. Lim, K.H. Noh, D. Park, H.-S. Cho, K. Susztak, C.-R. Jung, Effective reconstruction of functional organotypic kidney spheroid for in vitro nephrotoxicity studies, Scientific reports, 9 (2019) 17610.

[16] C. Luckert, C. Schulz, N. Lehmann, M. Thomas, U. Hofmann, S. Hammad, J.G. Hengstler, A. Braeuning, A. Lampen, S. Hessel, Comparative analysis of 3D culture methods on human HepG2 cells, Archives of toxicology, 91 (2017) 393-406.

[17] M.A. Tutty, G. Vella, A. Prina-Mello, Pre-clinical 2D and 3D toxicity response to a panel of nanomaterials; comparative assessment of NBM-induced liver toxicity, Drug delivery and translational research, 12 (2022) 2157-2177.

[18] L. Wu, D. Vllasaliu, Q. Cui, B.T. Raimi-Abraham, In situ self-assembling liver spheroids with synthetic nanoscaffolds for preclinical drug screening applications, ACS Applied Materials & Interfaces, 16 (2024) 25610-25621.

[19] J.-E. Seo, H. Xu, X. Li, A.H. Atrakchi, T. McGovern, K.L.D. Bruno, D.A. Keire, N. Mei, R.H. Heflich, X. Guo, Genotoxicity evaluation of ten nitrosamine drug substance-related impurities using 2D and 3D HepaRG cells, Regulatory Toxicology and Pharmacology, (2025) 105906.

[20] J.-E. Seo, J.Z. Yu, H. Xu, X. Li, A.H. Atrakchi, T.J. McGovern, K.L.D. Bruno, N. Mei, R.H. Heflich, X. Guo, Genotoxicity assessment of eight nitrosamines using 2D and 3D HepaRG cell models, Archives of toxicology, 97 (2023) 2785-2798.

[21] V. Zingales, M.R. Esposito, M. Quagliata, E. Cimetta, M.-J. Ruiz, Comparative study of spheroids (3D) and monolayer cultures (2D) for the in vitro assessment of cytotoxicity induced by the mycotoxins sterigmatocystin, ochratoxin a and patulin, Foods, 13 (2024) 564.

[22] Y. Zhong, J.S. Yu, X. Wang, B. Binas, H.H. Yoo, Chemical‐based primary human hepatocyte monolayer culture for the study of drug metabolism and hepatotoxicity: comparison with the spheroid model, The FASEB Journal, 35 (2021) e21379.

[23] L. Jamshed, S. Jamshed, R.A. Frank, L.M. Hewitt, P.J. Thomas, A.C. Holloway, Assessing receptor activation in 2D and 3D cultured hepatocytes: responses to a single compound and a complex mixture, Toxics, 12 (2024) 631.

[24] M. Saleh, K. Hogeveen, V. Fessard, L. Le Hégarat, Mechanistic toxicity profiling of chemicals: A comparative study in 2D and 3D HepaRG models using high-content analysis, Toxicology, (2025) 154280.

[25] B. Sun, Z. Liang, Y. Wang, Y. Yu, X. Zhou, X. Geng, B. Li, A 3D spheroid model of quadruple cell co-culture with improved liver functions for hepatotoxicity prediction, Toxicology, 505 (2024) 153829.

[26] V. Holgersson, S. Joyce, M. Brookman-Amissah, T. Lammel, Comparative analysis of 3D and 2D in vitro models of the permanent fish liver cell line RTL-W1: Metabolic capabilities and responses to xenobiotics, Ecotoxicology and Environmental Safety, 288 (2024) 117327.

[27] S. Xu, L. Ma, T. Wu, Y. Tian, L. Wu, Assessment of cellular senescence potential of PM2. 5 using 3D human lung fibroblast spheroids in vitro model, Toxicology Research, 13 (2024) tfae037.

[28] J. Laski, B. Singha, X. Wang, Y.R. Valdés, O. Collins, T.G. Shepherd, Activated CAMKKβ-AMPK signaling promotes autophagy in a spheroid model of ovarian tumour metastasis, Journal of Ovarian Research, 13 (2020) 58.

[29] A. Diane, L.I. Mohammed, H.H. Al-Siddiqi, Islets in the body are never flat: transitioning from two-dimensional (2D) monolayer culture to three-dimensional (3D) spheroid for better efficiency in the generation of functional hPSC-derived pancreatic β cells in vitro, Cell Communication and Signaling, 21 (2023) 151.

[30] X. Chao, F. Zhao, J. Hu, Y. Yu, R. Xie, J. Zhong, M. Huang, T. Zeng, H. Yang, D. Luo, Comparative study of two common in vitro models for the pancreatic islet with MIN6, Tissue engineering and regenerative medicine, 20 (2023) 127-141.

[31] A. Essaouiba, R. Jellali, M. Shinohara, B. Scheidecker, C. Legallais, Y. Sakai, E. Leclerc, Analysis of the behavior of 2D monolayers and 3D spheroid human pancreatic beta cells derived from induced pluripotent stem cells in a microfluidic environment, Journal of biotechnology, 330 (2021) 45-56.

[32] M.K. Wong, M. Wahed, S.A. Shawky, A. Dvorkin-Gheva, S. Raha, Transcriptomic and functional analyses of 3D placental extravillous trophoblast spheroids, Scientific reports, 9 (2019) 12607.

[33] M.K. Wong, S.A. Shawky, A. Aryasomayajula, M.A. Green, T. Ewart, P.R. Selvaganapathy, S. Raha, Extracellular matrix surface regulates self-assembly of three-dimensional placental trophoblast spheroids, PloS one, 13 (2018) e0199632.

[34] V. Stojanovska, S. Arnold, M. Bauer, H. Voss, S. Fest, A.C. Zenclussen, Characterization of three-dimensional trophoblast spheroids: an alternative model to study the physiological properties of the placental unit, Cells, 11 (2022) 2884.

[35] Y. Pan, L. Han, Y. Yang, X. Wu, A. Wang, L. Xie, W. Zhu, S. Wang, Y. Lei, Alternating 2D and 3D culture reduces cell size and extends the lifespan of placenta-derived mesenchymal stem cells, Frontiers in Bioengineering and Biotechnology, 13 (2025) 1632810.

[36] Y. Pan, L. Han, Y. Yang, X. Wu, A. Wang, L. Xie, W. Zhu, S. Wang, Y. Lei, Alternating 2D and 3D culture reduces cell size and extends the lifespan of placenta-derived MSCs, bioRxiv, (2025) 2025.2005. 2020.655182.

[37] A. Hawkins, P. Pantazi, L. Yang, C.B. Coyne, V. Bokun, J.M. Lemme-Dumit, M.F. Pasetti, S. Barnett, F.J. Culley, B. Holder, Long-term culture and passaging of term trophoblast for the investigation of syncytiotrophoblast function, Placenta, 166 (2025) 25-32.

[38] M.A. Sheridan, X. Zhao, R.C. Fernando, L. Gardner, V. Perez-Garcia, Q. Li, S.G. Marsh, R. Hamilton, A. Moffett, M.Y. Turco, Characterization of primary models of human trophoblast, Development, 148 (2021) dev199749.

[39] E. Lorza-Gil, O. Strauss, E. Ziegler, K. Kansy, M.-T. Katschke, G. Rahimi, D. Neuscheler, L. Sandforth, A. Sandforth, G. Sancar, Incretin-responsive human pancreatic adipose tissue organoids: a functional model for fatty pancreas research, Molecular Metabolism, 91 (2025) 102067.

[40] H. Rezanejad, J.H. Lock, B.A. Sullivan, S. Bonner‐Weir, Generation of Pancreatic Ductal Organoids and Whole‐Mount Immunostaining of Intact Organoids, Current protocols in cell biology, 83 (2019) e82.

[41] X. Guan, X. Chen, Y. Xu, L. Wang, Q. Zhang, X. Luo, Y. Yin, R. Zhang, X. Liu, F. Wan, Directed differentiation of human pluripotent stem cells into pancreatobiliary co-progenitor-like population, Biochemical and Biophysical Research Communications, (2025) 152563.

[42] J. Youk, T. Kim, K.V. Evans, Y.-I. Jeong, Y. Hur, S.P. Hong, J.H. Kim, K. Yi, S.Y. Kim, K.J. Na, Three-dimensional human alveolar stem cell culture models reveal infection response to SARS-CoV-2, Cell Stem Cell, 27 (2020) 905-919. e910.

[43] N. Sachs, A. Papaspyropoulos, D.D. Zomer‐van Ommen, I. Heo, L. Böttinger, D. Klay, F. Weeber, G. Huelsz‐Prince, N. Iakobachvili, G.D. Amatngalim, Long‐term expanding human airway organoids for disease modeling, The EMBO journal, 38 (2019) EMBJ2018100300.

[44] E. Eenjes, S. van Riet, A.A. Kroon, A.M. Slats, P.P.S. Khedoe, A. Boerema-de Munck, M. Buscop-van Kempen, D.K. Ninaber, I.K. Reiss, H. Clevers, Disease modeling following organoid-based expansion of airway epithelial cells, American Journal of Physiology-Lung Cellular and Molecular Physiology, 321 (2021) L775-L786.

[45] T. Takebe, K. Sekine, M. Enomura, H. Koike, M. Kimura, T. Ogaeri, R.-R. Zhang, Y. Ueno, Y.-W. Zheng, N. Koike, Vascularized and functional human liver from an iPSC-derived organ bud transplant, Nature, 499 (2013) 481-484.

[46] S.J. Mun, J.-S. Ryu, M.-O. Lee, Y.S. Son, S.J. Oh, H.-S. Cho, M.-Y. Son, D.-S. Kim, S.J. Kim, H.J. Yoo, Generation of expandable human pluripotent stem cell-derived hepatocyte-like liver organoids, Journal of hepatology, 71 (2019) 970-985.

[47] E. Kang, J.-W. Kim, S. Jo, J.H. Ryu, H. Noh, H.-J. Park, H. Kim, Comparative study of CYP450 gene regulation in human pluripotent stem cell-derived liver 2D cell and 3D organoid models, Toxicology, 517 (2025) 154209.

[48] Y. Tong, Y. Ueyama-Toba, H. Mizuguchi, Biliary epithelial cell differentiation of bipotent human liver-derived organoids by 2D and 3D culture, Biochemistry and Biophysics Reports, 33 (2023) 101432.

[49] S.M. Czerniecki, N.M. Cruz, J.L. Harder, R. Menon, J. Annis, E.A. Otto, R.E. Gulieva, L.V. Islas, Y.K. Kim, L.M. Tran, High-throughput screening enhances kidney organoid differentiation from human pluripotent stem cells and enables automated multidimensional phenotyping, Cell stem cell, 22 (2018) 929-940. e924.

[50] Y.S. Son, N. Son, W.D. Yu, A. Baek, Y.-J. Park, M.-S. Lee, S.-J. Lee, D.-S. Kim, M.-Y. Son, Particulate matter 10 exposure affects intestinal functionality in both inflamed 2D intestinal epithelial cell and 3D intestinal organoid models, Frontiers in immunology, 14 (2023) 1168064.

[51] Y.R. Lewis-Israeli, A.H. Wasserman, M.A. Gabalski, B.D. Volmert, Y. Ming, K.A. Ball, W. Yang, J. Zou, G. Ni, N. Pajares, Self-assembling human heart organoids for the modeling of cardiac development and congenital heart disease, Nature communications, 12 (2021) 5142.

[52] L. Drakhlis, S. Biswanath, C.-M. Farr, V. Lupanow, J. Teske, K. Ritzenhoff, A. Franke, F. Manstein, E. Bolesani, H. Kempf, Human heart-forming organoids recapitulate early heart and foregut development, Nature biotechnology, 39 (2021) 737-746.

[53] X. Wu, Y. Chen, A. Luz, G. Hu, E.J. Tokar, Cardiac development in the presence of cadmium: an in vitro study using human embryonic stem cells and cardiac organoids, Environmental Health Perspectives, 130 (2022) 117002.

[54] C. Brighi, F. Salaris, A. Soloperto, F. Cordella, S. Ghirga, V. De Turris, M. Rosito, P.F. Porceddu, C. D’Antoni, A. Reggiani, Novel fragile X syndrome 2D and 3D brain models based on human isogenic FMRP-KO iPSCs, Cell Death & Disease, 12 (2021) 498.

[55] L.M. Smits, L. Reinhardt, P. Reinhardt, M. Glatza, A.S. Monzel, N. Stanslowsky, M.D. Rosato-Siri, A. Zanon, P.M. Antony, J. Bellmann, Modeling Parkinson’s disease in midbrain-like organoids, NPJ Parkinson's disease, 5 (2019) 5.

[56] I. Slavin, S. Dea, P. Arunkumar, N. Sodhi, S. Montefusco, J. Siqueira-Neto, J. Seelke, M.A. Lofstrom, B. Anson, F. Zanella, Human iPSC-derived 2D and 3D platforms for rapidly assessing developmental, functional, and terminal toxicities in neural cells, International Journal of Molecular Sciences, 22 (2021) 1908.

[57] Y. Mei, D. Wu, J. Berg, B. Tolksdorf, V. Roehrs, A. Kurreck, T. Hiller, J. Kurreck, Generation of a perfusable 3D lung cancer model by digital light processing, International Journal of Molecular Sciences, 24 (2023) 6071.

[58] D. Wu, J. Berg, B. Arlt, V. Röhrs, M.A. Al-Zeer, H.E. Deubzer, J. Kurreck, Bioprinted cancer model of neuroblastoma in a renal microenvironment as an efficiently applicable drug testing platform, International journal of molecular sciences, 23 (2021) 122.

[59] B. Blanco-Fernandez, S. Rey-Vinolas, G.l.n. Bağcı, G. Rubi-Sans, J. Otero, D. Navajas, S. Perez-Amodio, E. Engel, Bioprinting decellularized breast tissue for the development of three-dimensional breast cancer models, ACS applied materials & interfaces, 14 (2022) 29467-29482.

[60] Y.T. Tung, Y.C. Chen, K. Derr, K. Wilson, M.J. Song, M. Ferrer, A 3D bioprinted human neurovascular unit model of glioblastoma tumor growth, Advanced Healthcare Materials, 13 (2024) 2302831.

[61] M.A. Sullivan, S. Lane, A. Volkerling, M. Engel, E.L. Werry, M. Kassiou, Three‐dimensional bioprinting of stem cell‐derived central nervous system cells enables astrocyte growth, vasculogenesis, and enhances neural differentiation/function, Biotechnology and Bioengineering, 120 (2023) 3079-3091.

[62] W. Chen, K. Gai, X. Luo, B. Wu, X. Wang, W. Shi, K. Zhang, F. Lin, W. Sun, Y. Song, 3D bioprinted in vitro epilepsy models for pharmacological evaluation in temporal lobe epilepsy, Biofabrication, 17 (2025) 015018.

[63] G.A. Salg, E. Poisel, M. Neulinger-Munoz, J. Gerhardus, D. Cebulla, C. Bludszuweit-Philipp, V. Vieira, F. Nickel, I. Herr, A. Blaeser, Toward 3D-bioprinting of an endocrine pancreas: A building-block concept for bioartificial insulin-secreting tissue, Journal of Tissue Engineering, 13 (2022) 20417314221091033.

[64] K.J. Wolf, R.C. van Gaal, S.G. Uzel, J.E. Rubins, A.N. Klaus, A. Printz, P. Nair, K.T. Kroll, P. Stankey, L.M. Satlin, Perfusable 3D models of ureteric bud and collecting duct tubules, Cell Biomaterials, 2 (2026).

[65] F. Perin, A. Ricci, S. Fagiolino, A. Rak-Raszewska, H. Kearney, J. Ramis, I. Bereen, M. Baker, D. Maniglio, A. Motta, Bioprinting of Alginate-Norbornene bioinks to create a versatile platform for kidney in vitro modeling, Bioactive Materials, 49 (2025) 550-563.

[66] R. Pichler, L. Rizzo, K. Tröndle, M. Bühler, H. Brucker, A.-L. Müller, K. Grand, S. Fare, A. Viau, M.M. Kaminski, Tuning the 3D microenvironment of reprogrammed tubule cells enhances biomimetic modeling of polycystic kidney disease, Biomaterials, 291 (2022) 121910.

[67] K.A. Gold, B. Saha, N.K. Rajeeva Pandian, B.K. Walther, J.A. Palma, J. Jo, J.P. Cooke, A. Jain, A.K. Gaharwar, 3D bioprinted multicellular vascular models, Advanced healthcare materials, 10 (2021) 2101141.

[68] M. De Villiers, L.H. Du Plessis, Flow cytometry as an analytical method of drug-induced apoptosis in 3D bioprinted melanoma cells, Biomedical Materials, 18 (2023) 045031.

[69] N. Moghimi, S.A. Hosseini, A.B. Dalan, D. Mohammadrezaei, A. Goldman, M. Kohandel, Controlled tumor heterogeneity in a co-culture system by 3D bio-printed tumor-on-chip model, Scientific Reports, 13 (2023) 13648.

[70] L. Grunewald, T. Lam, L. Andersch, A. Klaus, S. Schwiebert, A. Winkler, A. Gauert, A.I. Heeren-Hagemann, K. Astrahantseff, F. Klironomos, A reproducible bioprinted 3D tumor model serves as a preselection tool for CAR T cell therapy optimization, Frontiers in immunology, 12 (2021) 689697.

[71] F. Meng, C.M. Meyer, D. Joung, D.A. Vallera, M.C. McAlpine, A. Panoskaltsis‐Mortari, 3D bioprinted in vitro metastatic models via reconstruction of tumor microenvironments, Advanced Materials, 31 (2019) 1806899.

[72] M. Chirivì, C. Bearzi, P. Rosa, S. Miglietta, F. Petronella, E. De Falco, A. Calogero, R. Pani, V. Petrozza, G. Perotto, Biomimetic keratin-coated gold nanoparticles for photo-thermal therapy in a 3D bioprinted glioblastoma tumor model, International journal of molecular sciences, 23 (2022) 9528.

[73] C. Richards, H. Chen, M. O’Rourke, A. Bannister, G. Owen, A. Volkerling, A. Ghosh, C.A. Gorrie, D. Gallego-Ortega, A.L. Bottomley, Matrix directs trophoblast differentiation in a bioprinted organoid model of early placental development, Nature Communications, 16 (2025) 8267.
